# Methanol consumption drives the bacterial chloromethane sink in a forest soil

**DOI:** 10.1038/s41396-018-0228-4

**Published:** 2018-07-10

**Authors:** Pauline Chaignaud, Mareen Morawe, Ludovic Besaury, Eileen Kröber, Stéphane Vuilleumier, Françoise Bringel, Steffen Kolb

**Affiliations:** 1 Department of Microbiology, Genomics and the Environment, Université de Strasbourg, CNRS, GMGM UMR 7156, Strasbourg, France; 2 Department of Ecological Microbiology, University of Bayreuth, Bayreuth, Germany; 3 Microbial Biogeochemistry, RA Landscape Functioning, ZALF Leibniz Centre for Landscape Research, Müncheberg, Germany; 4 Present Address: UMR FARE 614 Fractionnement des AgroRessources et Environnement, Chaire AFERE, INRA, Université de Reims Champagne-Ardenne, Reims, France

## Abstract

Halogenated volatile organic compounds (VOCs) emitted by terrestrial ecosystems, such as chloromethane (CH_3_Cl), have pronounced effects on troposphere and stratosphere chemistry and climate. The magnitude of the global CH_3_Cl sink is uncertain since it involves a largely uncharacterized microbial sink. CH_3_Cl represents a growth substrate for some specialized methylotrophs, while methanol (CH_3_OH), formed in much larger amounts in terrestrial environments, may be more widely used by such microorganisms. Direct measurements of CH_3_Cl degradation rates in two field campaigns and in microcosms allowed the identification of top soil horizons (i.e., organic plus mineral A horizon) as the major biotic sink in a deciduous forest. Metabolically active members of *Alphaproteobacteria* and *Actinobacteria* were identified by taxonomic and functional gene biomarkers following stable isotope labeling (SIP) of microcosms with CH_3_Cl and CH_3_OH, added alone or together as the [^13^C]-isotopologue. Well-studied reference CH_3_Cl degraders, such as *Methylobacterium extorquens* CM4, were not involved in the sink activity of the studied soil. Nonetheless, only sequences of the *cmuA* chloromethane dehalogenase gene highly similar to those of known strains were detected, suggesting the relevance of horizontal gene transfer for CH_3_Cl degradation in forest soil. Further, CH_3_Cl consumption rate increased in the presence of CH_3_OH. Members of *Alphaproteobacteria* and *Actinobacteria* were also ^13^C-labeled upon [^13^C]-CH_3_OH amendment. These findings suggest that key bacterial CH_3_Cl degraders in forest soil benefit from CH_3_OH as an alternative substrate. For soil CH_3_Cl-utilizing methylotrophs, utilization of several one-carbon compounds may represent a competitive advantage over heterotrophs that cannot utilize one-carbon compounds.

## Introduction

Chloromethane (CH_3_Cl) is the most abundant halogenated volatile organic compound (VOC) in the atmosphere and contributes substantially to destruction of stratospheric ozone [1, 2]. Global emissions reach about 2.0 ± 0.6 Tg CH_3_Cl per year [3, 4]. Current knowledge also suggests that terrestrial CH_3_Cl emissions are mainly associated with biological activities of the aboveground part of plants and with white rot fungi in soil [5–7]. CH_3_Cl is produced upon burning of plant biomass, from methoxy groups of plant structural components such as lignin and pectin [8, 9]. CH_3_Cl is also enzymatically produced by S-adenosylmethionine-dependent methylation of chloride [10]. Current estimates for the global sink are larger than the global source [3], to which forest soil could contribute as much as 1.0 Tg per year [3, 11]. Global budgets are still uncertain, as the nature of the biotic sink activity as well as its spatial and temporal variability are not known at the regional and global scale [12].

Net emissions of CH_3_Cl from terrestrial ecosystems are mitigated by soil and phyllosphere bacteria that utilize this VOC as a growth substrate [7, 13]. Such bacteria are aerobic methylotrophs, metabolically specialized for growth with one-carbon compounds [7]. The concentration of CH_3_Cl in the atmosphere is low (approx. 590 ppt). This suggests similar concentrations of CH_3_Cl in soils, although no experimental data are available to date [3, 14]. Previous studies suggest that aerobic soil methylotrophs can utilize CH_3_Cl at environmentally relevant nanomolar to picomolar concentrations [15–20]. Such low concentrations likely do not yield sufficient energy for substantial bacterial growth with CH_3_Cl. However, many known alphaproteobacterial CH_3_Cl degraders also grow with methanol (CH_3_OH) [13, 21], and this is also true in situ for soil methylotrophs of a deciduous forest [22]. Abundance of methylotrophs in O and A soil horizons is high, and ranges from 10^6^ to 3 × 10^8^ cells g_soil_^−1^, consistent with their frequent isolation from soils [23, 24].

Which organisms define the bacterial CH_3_Cl sink in soils is largely unknown at present. The only biochemically characterized pathway for CH_3_Cl utilization is the *cmu* pathway, characterized in detail for *Methylobacterium extorquens* CM4 [21]. It has been found in various CH_3_Cl-degrading bacterial strains, including several strains from forest soil [7, 18, 25–27]. The chloromethane dehalogenase gene *cmuA* has been used to detect CH_3_Cl-degraders in various environments [15, 16, 18, 28–30]. Nonetheless, there is experimental and genomic evidence that the *cmu* pathway is not the only pathway for CH_3_Cl utilization [13].

We hypothesized that (a) top soil (i.e., organic layer [O_f_, O_h_] plus A horizon) is the preferable habitat for CH_3_Cl degraders, and (b) that the as yet largely unknown CH_3_Cl bacterial sink in soils benefits from additional energy and carbon sources, such as CH_3_OH as a widespread methylotrophic growth substrate in soil [23, 24]. These hypotheses were tested for a European deciduous forest dominated by beech (*Fagus sylvatica* L.), and in a multi-treatment DNA stable isotope probing (SIP) laboratory experiment involving combinations of ^13^C-labeled and unlabeled CH_3_OH and CH_3_Cl amendment. Amplicon sequencing of 16S rRNA, *cmuA*, and *mxaF*/*xoxF* genes as relevant gene markers was employed to identify microorganisms potentially involved in the CH_3_Cl sink.

## Materials and methods

Sampling site. Samples were collected in the natural forest reserve area Steigerwald located in South Germany (49° 37′ N, 10° 17′ O; sandy loam; Dystric Cambisol; pH of 4.6; mean annual temperature 7.5 °C; mean annual precipitation 725 mm). Steigerwald forest has not been managed for at least 25 years and represents a quasi-pristine deciduous forest typical of Central Europe, i.e., predominance of European beech (*Fagus sylvatica* L.) with minor stocks of oak (*Quercus robur* L.) [31].

### Field closed-chamber measurements of CH_3_Cl consumption

Eight closed top chambers were installed on-site at 12 am on 19 June 2013 and 20 August 2013. Air temperatures were 19 °C and 23 °C, respectively. The self-constructed stainless steel chambers had a total volume of 25.1 L (40 cm diameter; 20 cm height) and were equipped with an injection port. Chambers were spiked with 100 ppm CH_3_Cl [32] and gas samples were collected over a period of 180 min with gas-tight syringes, and stored in exetainers (Labco Limited, England) for subsequent analysis by gas chromatography (GC).

### First order rate constants of CH_3_Cl-degradation

Fresh beech leaves, dead leaf litter, soil horizons (O_f_, A_h_, B), and rotting wood were investigated. For beech phyllosphere analysis, fresh beech leaves were sampled by cutting branches from trees and a pool of cut leaves were immediately used for experiments. Samples were taken on 20 August 2013 (8 locations per compartment pooled) and on 12 July 2016 (3 locations pooled), and transferred to duplicated gas-tight 125 mL serum bottles that were subsequently flushed with synthetic air (Rießner Gase GmbH, Germany). CH_3_Cl (Rießner Gase GmbH, Germany, purity 99.99%) was injected to final concentrations of 60 ppb or 200 ppm for samples taken on 20 August 2013 and 12 July 2016, respectively. Controls included a substrate-free control, a biological process control (amended with 20 mM KCN to inhibit biological activity), and an anoxic control (headspace was pure N_2_). All treatments and controls were performed in triplicate. Gas samples were taken with gas-tight syringes and stored in 3 mL, pre-evacuated exetainers (Labco ltm, UK) for further analysis by gas chromatography.

### Soil microcosm set-up for DNA stable isotope probing (DNA SIP)

Five samples from the upper soil layer within a 20 m circle, were taken on 20 August 2013, pooled, homogenized and sieved (2 mm-mesh size). To stimulate CH_3_Cl consumption, sieved soil was aliquoted in batches of 500 g into sealed flasks, CH_3_Cl (Sigma Aldrich) added to the head space at a final concentration of 1%, and incubated in 1 L glass flasks at 20°C in the dark. Mixing ratios of CH_3_Cl were followed by GC until added CH_3_Cl was consumed. Then, for each microcosm of the DNA SIP experiment, 70 g activated soil was transferred to a 500 mL flask sealed with a Viton stopper. Eight different treatments were prepared, each performed in duplicates. Four treatments were amended with ^13^C-labeled substrates and four with unlabeled ^12^C-substrates. [^13^C]-CH_3_Cl (Sigma Aldrich), [^13^C]-CH_3_Cl together with unlabeled CH_3_OH (Campro Scientific), [^13^C]-CH_3_OH (Sigma Aldrich) together with unlabeled CH_3_Cl, and [^13^C]-CH_3_OH (Table 1). Control incubations were amended with equivalent amounts of unlabeled substrates, or left without amendment (Table 1). CH_3_Cl and/or CH_3_OH were amended as unlabeled or ^13^C-labeled isotopologue in 5 pulses over a period of 23 days (Fig. S1). CH_3_Cl and CO_2_ headspace mixing ratios were monitored by GC, and amended again when CH_3_Cl was no longer detectable. After each pulse, 5 soil aliquots (1 g each) per microcosm were retrieved and stored at −80 °C until further analysis.

**Table 1:** Microcosm setup and carbon substrate amendment

Carbon source added	Total carbon added (mM)^a^	Gas phase amendment^b^	Liquid phase amendment^c^
[^13^C]-CH_3_Cl	18	[^13^C]-CH_3_Cl	H_2_O
[^13^C]-CH_3_Cl and CH_3_OH	36	[^13^C]-CH_3_Cl	CH_3_OH
CH_3_Cl and [^13^C]-CH_3_OH	36	CH_3_Cl	[^13^C]-CH_3_OH
[^13^C]-CH_3_OH	18	–	[^13^C]-CH_3_OH
CH_3_Cl	18	CH_3_Cl	H_2_O
CH_3_Cl and CH_3_OH	36	CH_3_Cl	CH_3_OH
CH_3_OH	18	–	CH_3_OH
Control	–	–	H_2_O

^a^Added per pulse (5 pulses in total)

^b^A volume of 6 mL CH_3_Cl was added for each CH_3_Cl pulse. At each pulse, a total gas phase volume of 56 mL (air with or without CH_3_Cl) was added to 500 mL flasks in order to maintain overpressure in the flasks

^c^Either 1 mL milliQ water or 216 mM CH_3_OH stock solution was added per pulse to each microcosm

### pH and gravimetric water content in soil samples

pH was measured on sieved soil, before activation and after each substrate pulse using an InLab R422 pH electrode (InLab Semi-Micro; Mettler-Toledo, Gießen, Germany). Gravimetric soil moisture content was determined by weighing the soil before and after weight constancy following drying at 60 °C.

### Quantification of CH_3_Cl, CO_2_, and ^13^CO_2_

On 19 June 2013 and 20 August 2013, CH_3_Cl and CO_2_ mixing ratios were determined by GC (HP 5890 Series II, Agilent) using a Porapak Q 80/100 column (Supelco, USA) and a helium-methane mixture (95:5) as the carrier gas. On 16 July 2016, CH_3_Cl mixing ratios were determined by ISQ^TM^ Quadrupole mass spectrometer (MS) coupled with TRACE^TM^ Ultra gas chromatograph (GC) (Thermo Fisher Scientific, Massachusetts, USA). CH_3_Cl and CO_2_ mixing ratios were calculated by regression analysis based on a 5-point calibration with standard mixing ratios of both gases. In the SIP experiment, GC MS analysis (Perkin–Elmer GC Clarus 600 system) was carried out as described previously [22]. Further Details of gas analysis methods are given in Supplemental Information.

### Nucleic acid extraction and RNA removal

Nucleic acids from experimental replicates of all treatments and controls were extracted from each 0.5 g of soil when CH_3_Cl had been consumed after the third substrate pulse [33]. RNA was removed according to standard procedures including a treatment with RNAse followed by isopropanol precipitation (Supplemental Information). Recovered DNA was resuspended in DNAse-free water, quantified, and eventually stored at −80 °C until further processing through isopycnic centrifugation [22].

### DNA fractionation by isopycnic centrifugation

Separation of the heavy (H) and light (L) DNA in a cesium chloride gradient was described in detail previously [22, 34]. In brief, a cesium chloride gradient solution mixed with each RNA-free DNA was loaded into an ultracentrifugation tube, placed in a Vti 65.2 vertical rotor (Beckman Coulter, Germany) and centrifuged for 40 h at 177,000×*g* in a LE-70 ultracentrifuge (Beckman Coulter, Germany). DNA was harvested according to established procedures in 10 gradient fractions [22, 34]. The main four fractions of H and of L DNA were pooled and DNA was quantified [22] (Fig. S2a-b, Supplemental Information). DNA concentrations ranged between 0.3 and 3.0 ng µL^−1^ for H fractions, and 18.5 and 50.3 ng µL^−1^ for L fractions.

### PCR amplification, high-throughput sequencing, and data processing

PCR amplification of the 16S rRNA gene, and of CH_3_Cl dehalogenase CmuA and methanol dehydrogenase MxaF/XoxF encoding genes are described in detail in Table 2, and in Table S2 (Supplemental Information). New primers were designed to detect a larger spectrum of genetic diversity using NGS sequencing both for *cmuA* (Table S2; Fig. S7) and *mxaF* / *xoxF* (Supplemental Information, Table S2, Fig S8). For sequencing analysis following PCR amplification, briefly, a barcode oligonucleotide identifying sample origin was ligated to each PCR product. Equimolar pools of all libraries were assembled, and the resulting combined library was sequenced using Illumina MiSeq technology (LGC Genomics GmbH, Germany). Reads were assembled into contigs and analyzed using Mothur v.1.33.2 with default parameters (http://www.mothur.org/wiki/MiSeq_SOP) [35]. 16S rRNA reads <420 or >460 bp were discarded. Reads were pre-clustered into groups of sequences with up to 2 nucleotide differences. Chimera sequences were removed with UCHIME [36]. Remaining sequences were assigned by naïve Bayesian taxonomic classification using the SILVA reference database. Sequences that could not be assigned to *Bacteria* and *Archaea* were excluded from further analysis. OTUs were defined at 98% sequence similarity. Raw reads of *cmuA* and *mxaF*/*xoxF* amplicons with read lengths within 20 nucleotides of the expected amplicon length were clustered by USEARCH [37]. Sequences occurring only once in all libraries were considered artefactual and removed, but singletons within individual amplicon libraries were kept. Reads were clustered iteratively at progressively lower cut-off values, and the cut-off value at which the number of retrieved OTUs stabilized was selected [38]. These OTU sequences were compared against a gene-specific database generated from GenBank using BLAST (http://blast.ncbi.nlm.nih.gov) for identification. Taxonomic assignments of consensus sequences of each OTU were used to identify ^13^C-labeled gene OTUs.

**Table 2:** Analyzed gene markers and amplicon characteristics

Gene marker	Function	PCR Primer	Sequence (5′-3′)^a^	Amplicon size (bp)	SNP/ OTU^b^	Total OTUs^c^	Labeled OTUs^d^	Reference
16 S rRNA gene	Ribosomal small subunit RNA	341for	CCTACGGGNGGCWGCAG	464	9	117	5	[60]
		785/805rev	GACTACHVGGGTATCTAATCC					[61]
*cmuA*	chloromethane methyltransferase	cmuAf422	GARGTBGGITAYAAYGGHGG	422	38	8	5	This study^f^
		cmuAr422	TCRTTGCGCTCRTACATGTCICC					
*mxaF/xoxF* ^e^	methanol dehydrogenase	mdh1	GCGGIWSCAICTGGGGYT	430	39	6	6	This study^f^
		mdh2	GCGGIWSGAICTGGGGYT					
		mdhR	GAASGGYTCSYARTCCATGCA					

^a^Degenerate base mixtures: B (C,G,T), H (A,C,T), K (G,T), N (A,C,G,T), R (A,G), S (G,C), Y (C,T), V (A, C, T), W (A,T). Inosine (I) was used instead of the N mixture [62] in some cases

^b^Maximal Single Nucleotide Polymorphism (SNP) positions possible within an OTU

^c^Corresponding to the sum of OTUs detected in the 8 microcosms of the SIP experiment. Sequences were affiliated to the same OTU at cutoff values of 98, 90, and 80% sequence identity at the nucleotide level for 16S rRNA gene, *cmuA*, and *mxaF/xoxF* amplicons, respectively

^d^See Material and Methods for the criteria applied to define OTUs as ‘labeled’

^e^Primer pairs allow to amplify both *mxaF* and *xoxF* types of methanol dehydrogenase (mdh) sub units Amplifications were performed with two different forward primers (mdh1, mdh2) in order to reduce primer degeneracy and improve PCR efficiency. Amplicons obtained with primers mdh1/mdhR and mdh2/mdhR were pooled before sequencing.

^f^See Supplemental Information of Materials and Methods for details

### Identification of ^13^C-labeled OTUs

Labeled OTUs were defined according to a previously reported protocol developed to minimize false positives in ‘H DNA’ fractions [22, 39]. The following criteria were applied to identify ^13^C-labeled OTUs: (1) OTU abundance in the ‘heavy’ fraction of a microcosm treated with ^13^C-labeled substrate higher than in the ‘heavy’ fraction of the corresponding microcosm treated with unlabeled substrate; (2) OTU abundance in the ‘heavy’ fraction higher than in the ‘light’ fraction; (3) OTU abundance in the ‘heavy’ fraction of the microcosm treated with ^13^C-labeled substrate ≥0.5%; (4) OTU abundance difference between ‘heavy’ and ‘light’ fractions higher by at least 0.3% (a threshold that considers the variance of each OTU abundance). Moreover, a lower limit of 5% was set for the labeling proportion (LP), (i.e., the relative frequency of a labeled OTU in a specific heavy fraction) of a given OTU to be considered as labeled [22].

### Statistical and phylogenetic analyzes

Richness and Simpson diversity indices were determined using Mothur and PAST (http://folk.uio.no/ohammer/past). Relationships between sequence datasets in different DNA fractions and microcosms were investigated by two-dimensional NMDS (non-metric multidimensional scaling) within Mothur, and visualized with Kaleidagraph (Synergy Software, Reading, PA, USA). Details on phylogenetic tree construction are given in figure legends.

### Nucleotide sequence accession numbers

Sequence datasets were deposited to the NCBI BioSample database under the study accession number SUB3319582.

## Results

**Fig. 1:**
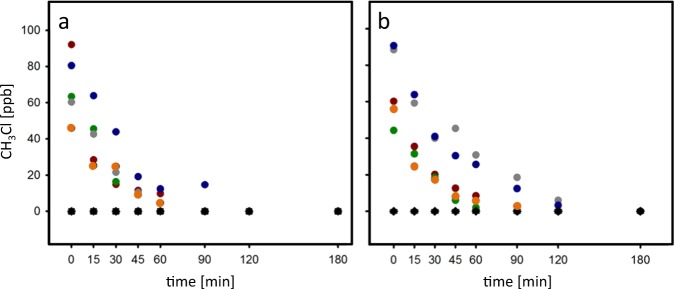
Dissipation of added CH_3_Cl in forest soil. Closed top chambers applied onto soil at the Steigerwald forest sampling site were amended with 100 ppm CH_3_Cl (color symbols, chambers 1–5) or incubated without supplementation (black symbols, triplicate controls) on **a** 19 June 2013 and **b** 20 August 2013

Localization of active CH_3_Cl consumption in different forest compartments. Two sampling campaigns were performed, with the second campaign aiming to verify and justify that top soil samples were indeed the most active in CH_3_Cl degradation, since only this horizon was selected to be assessed by SIP. Immediate consumption of CH_3_Cl was observed in two field site campaigns at the temperate deciduous forest Steigerwald (Fig. 1a, b). In contrast, no net CH_3_Cl emissions were detected from the forest floor (<1 ppb in chambers headspace). This strongly suggested that the investigated forest top soil represents a major sink for atmospheric CH_3_Cl at the forest ecosystem level.

**Fig. 2:**
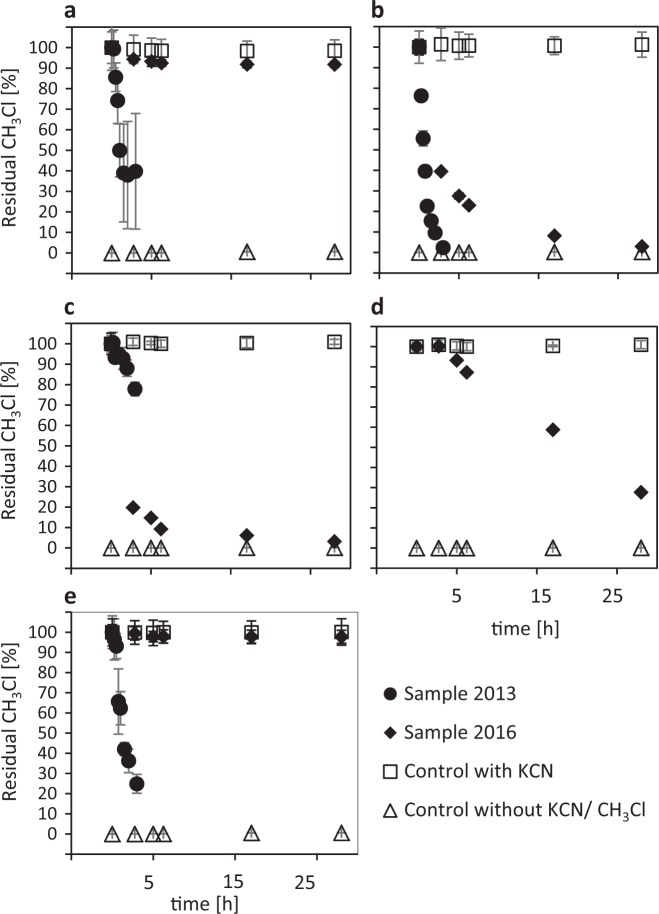
Dissipation of added CH_3_Cl in microcosms of forest compartments. First order rate constants [k] of CH_3_Cl-amended microcosms of Steigerwald forest sampled on 20 August 2013 (circles) and on 12 July 2016 (diamonds). **a** Leaf litter (2013, *k* = 2.35 h^−1^; 2016, *k* = 0.19 h^−1^); **b** O_f_ horizon (2013, *k* = 3.04 h^−1^; 2016, *k* = 2.42 h ^−1^); **c** A_h_ horizon (2013, *k* = 6.93 h^−1^; 2016, *k* = 2.00 h^−1^); **d** B horizon (2016, *k* = 2.72); **e** fresh beech leaves (2013, k = 2.76 h^−1^; 2016, k = 0.06 h^−1^). Control experiments without amendment (triangles) or including KCN on top of CH_3_Cl to inhibit biological activity (squares) were also performed. B horizon was only measured in 2016

Results from lab-scale microcosms suggest that net CH_3_Cl consumption was primarily due to biological activity. Indeed, CH_3_Cl consumption was not observed in microcosms that were treated with KCN (Fig. 2a–e). O_f_ and A_h_ horizons were the most active layers (Fig. 2b, c). Because no CH_3_Cl consumption was detected under anoxic conditions (data not shown), top soil aerobic microorganisms likely represent the active sink for CH_3_Cl (Fig. 2b). Results obtained for the phyllosphere of European beech were variable, as we detected substantial consumption of CH_3_Cl in 2013 only (Fig. 2e).

### Mineralization and assimilation of CH_3_Cl by the top soil microbial community

As expected, a net increase in CO_2_ formation occurred in all microcosms in response to substrate amendment (Fig. S1a-c). A non-significant trend was observed towards larger CO_2_ release upon addition of CH_3_OH compared to CH_3_Cl (Fig. S1c). Rates of CO_2_ production were in the range of 0.2–0.3 mmol g_dry soil_^−1^ day^−1^ for microcosms to which CH_3_Cl or CH_3_OH was added. No differences in CO_2_ release between microcosms to which labeled or unlabeled substrates were added were observed, confirming that different carbon isotopologues did not affect carbon metabolism (*t*-test, *p* > 0.10; Fig. S1). Uncertainties were large, but about 10 mM (20%) of the added 54 mM of [^13^C]-CH_3_Cl were converted to [^13^C]-CO_2_ (Fig. S1). Hence, up to 80% of amended [^13^C]-CH_3_Cl was assimilated into biomass. This rather high rate may be an overestimate, given measurement uncertainties and undetected losses by precipitation of carbonate. An non-significant increase in [^13^C]-CO_2_ formation from labeled [^13^C]-CH_3_Cl was observed when unlabeled CH_3_OH was also provided (*t*-test, *p* > 0.10; Fig. S1c). In the reverse case of microcosms amended with [^13^C]-CH_3_OH together with unlabeled CH_3_Cl, the increase in [^13^C]-CO_2_ formation was slightly less. Moreover, CH_3_OH was mineralized to a larger extent than CH_3_Cl when added alone (*t*-test, *p* ≤ 0.05). Taken together, these observations suggest that microbial CH_3_Cl consumption activity is enhanced by CH_3_OH.

### Overall microbial community response to amended substrates

Based on three gene markers, amendment of substrates led to significant changes in microbial community composition, basing on statistical analyzes combining ^13^C-labeled and unlabeled phylotypes (Fig. S3a–c, Table S3, Tables 3 and 4). Combined with the observation that CO_2_ formation increased in amended microcosms (Fig. S1), this suggested increased growth of specific microorganisms with amended substrates.

**Table 3:** Number of filtered sequences obtained from heavy DNA fractions

Treatment	*16S rRNA* gene	*cmuA*	*mxaF/xoxF*
[^13^C]-CH_3_Cl	46110	4282	786
[^13^C]-CH_3_Cl and CH_3_OH	178042	3778	1534
CH_3_Cl and [^13^C]-CH_3_OH	76690	614	918
[^13^C]-CH_3_OH	98332	1886	588
CH_3_Cl	72522	370	2616
CH_3_Cl and CH_3_OH	47236	922	862
CH_3_OH	90822	246	2946
Unamended control	100032	184	2710

See Table S1 for further information

**Table 4:** Diversity indices for 16S rRNA gene OTUs obtained from heavy and light fractions of SIP experiment

Microcosm	SIP fraction	Sobs^a^	Shannon index^a^	Simpson diversity^a^
[^13^C]-CH_3_Cl	H	1762	5.95	44
	L	2870	7.82	223
[^13^C]-CH_3_Cl and CH_3_OH	H	1629	6.11	36
	L	3150	7.55	354
CH_3_Cl and [^13^C]-CH_3_OH	H	1566	6.52	120
	L	3062	7.86	283
[^13^C]-CH_3_OH	H	1215	4.58	7
	L	3148	7.40	322
Unamended control	H	1804	6.45	102
	L	2904	7.39	217

^a^Calculated at the 98% sequence identity level. See Materials and Methods for definitions. Simpson diversity is considered a conservative measure of the effective number of phylotypes [63]

### Diversity of ^13^C-labeled family-level OTUs based on the 16S rRNA gene

**Fig. 3:**
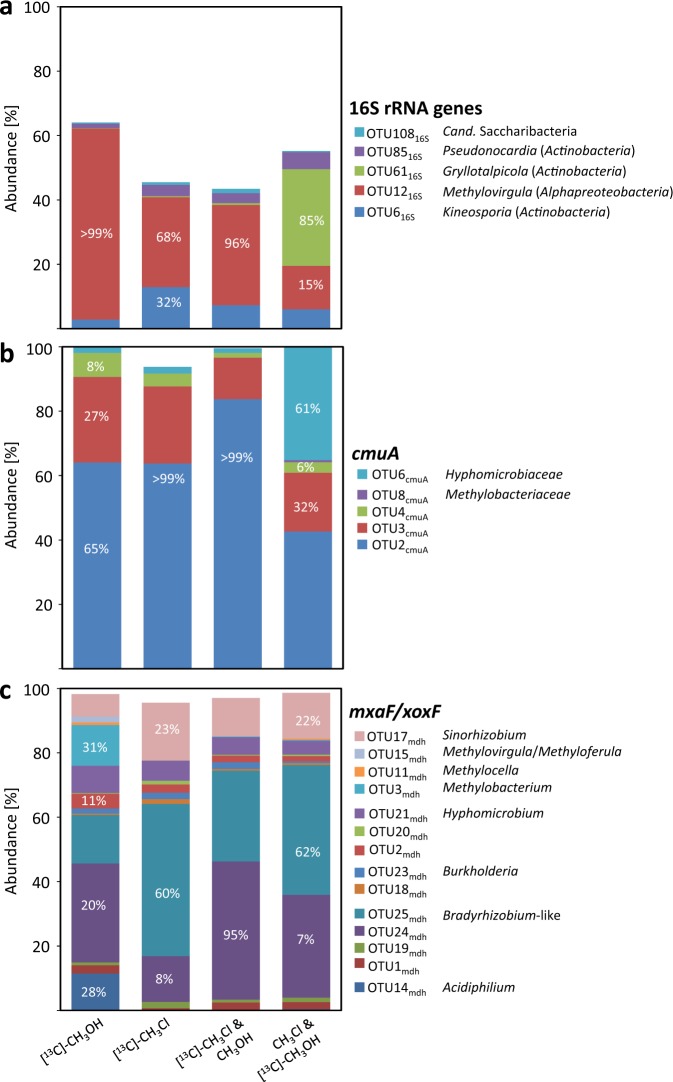
Relative abundance of [^13^C]-labeled phylotypes. **a** Bacterial 16S rRNA genes, **b** *cmuA*, and **c** *mxaF/xoxF*. Only microcosms exposed to [^13^C]-labeling are shown. Specific OTUs are indexed with corresponding gene markers (e.g., OTU108_16S_). Phylogenetic assignments are based on phylogenetic reconstructions (Fig. S3 [16S rRNA gene], Fig. S4 [*mxaF*/*xoxF*], and Fig. 4 [*cmuA*]). OTUs with >0.5% relative abundance in the H fraction but <5% labeling proportion were not considered as labeled. Unlabeled OTUs with relative abundance <0.5% are not shown

A total of 117 family-level OTUs (90% similarity) were detected (Table S1). Of these and across all four substrate treatments (Fig. 3a), several genera within five families were identified that satisfied the criteria set for defining ^13^C-labeled OTUs (Fig. 3a): *Beijerinckiaceae* within *Alphaproteobacteria*, three families of *Actinobacteria* (*Acidothermaceae*, *Pseudonocardiaceae*, and *Streptomycetaceae*) and OTU108_16S_ within the TM7 phylum, recently renamed as *Candidatus* Saccharibacteria (Fig. S4) [40]. *Methylovirgula*, *Acidothermus*, and *Streptomyces* represented over 95% of the *Beijerinckiaceae*, *Acidothermaceae*, and *Streptomycetaceae*, respectively (Fig. 3a). In the microcosm amended with [^13^C]-CH_3_Cl, three of the five family-level OTUs (*Acidothermaceae*, *Beijerinckiaceae*, and *Streptomycetaceae*) represented about 80% of all labeled OTUs (Fig. 3a). In the microcosm amended with [^13^C]-CH_3_OH, in contrast, mainly *Beijerinckiaceae* was labeled. The labeled *Cand*. Saccharibacteria-like OTU108_16S_ and the *Pseudonocardiaceae* sp.-like OTU85_16S_ were both found at low abundance in all ^13^C-amended microcosms (Fig. 3a). All labeled OTUs differed from the closest type strains as well as from previously described CH_3_Cl-degrading isolates (Fig. S4). Another interesting observation was that several other detected *Actinobacteria*, i.e. *Gryllotalpicola*, likely represent CH_3_OH utilizers based on labeling patterns (Fig. 3a, Fig S4).

### Diversity of CH_3_Cl utilizers based on *cmuA*

**Fig. 4:**
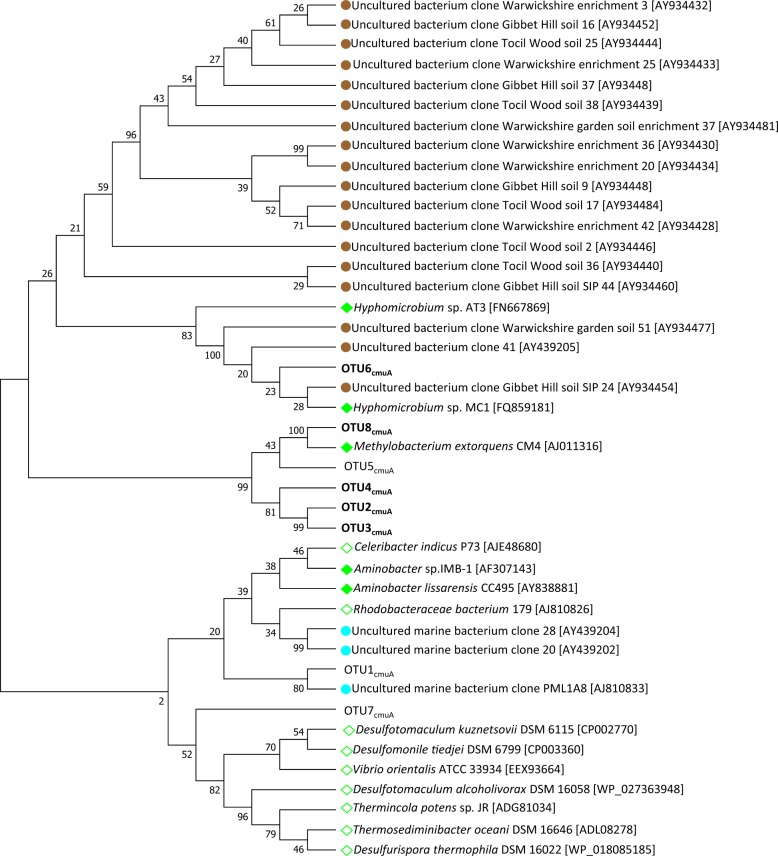
Phylogenetic affiliations of detected *cmuA* OTUs. A maximum likelihood phylogenetic tree was reconstructed from a 422 nt-long sequence alignment based on the Tamura-Nei model [59]. Bold, labeled *cmuA* OTUs. Scale bar, 0.05 substitutions per site. Bootstrapping was performed with 1000 replicates. Reference sequences from previous studies were included: characterized CH_3_Cl-utilizing isolates (green diamonds); uncharacterized genome-sequenced isolates containing *cmu* genes (green open diamonds); sequences detected by SIP in the marine environment (blue circles [30]) or in soil (brown circles [18])

Chloromethane:cobalamin methyltransferase *cmuA*, the biomarker for CH_3_Cl consumption by the *cmu* pathway, was amplified with newly designed primers (Table S2). Eight OTUs were detected among which five satisfied the criteria defined for ^13^C-labeled OTUs (Table S3). Consensus sequences of these 8 OTUs were compared to known *cmuA* of genome-sequenced cultivated strains, and of uncultivated OTUs identified in a previous SIP study of soil amended with [^13^C]-CH_3_Cl [15]. The eight OTUs belonged to three distinct gene clusters (Fig. 4). Labeled OTUs were closely related to sequences of known CH_3_Cl-degraders including *Methylobacterium extorquens* CM4 (>99 % identity) or *Hyphomicrobium* sp. MC1, as well as with *cmuA* sequences retrieved from a soil environment [15]. In the CH_3_Cl treatment, OTU2_cmuA_ and OTU3_cmuA_ were among the most dominant ^13^C-labeled OTUs. This labeling pattern was conserved in all ^13^C-labeled substrate treatments of our study (Fig. 3b). However, when methanol was the ^13^C-labeled substrate, an additional OTU6_cmuA_ was detected (Fig. 3b). OTU6_cmuA_ was very similar to *cmuA* genotypes of reference CH_3_Cl-degrading *Hyphomicrobium* strains (Fig. 4). All other ^13^C-labeled OTUs represented minor populations with closest similarity to *cmuA* from *Methylobacterium* (Figs. 3b, 4).

### Diversity of methanol utilizers based on *mxaF/xoxF*

Two types of methanol dehydrogenase (mdh) are predominantly found in methanol utilizers, MxaFI and XoxF, both harboring a pyrroloquinoline quinone catalytic center [41]. Previously used *mxaF*/*xoxF* primers were biased against *xoxF* [42]. Thus, we designed new *mdh* primers targeting both types of MDH (Table 2 and S2; Fig. S8). A total of 15 distinct ^13^C-labeled *mdh* OTUs were found, all but one *xoxF* genes (Figs. 3c and S5). These OTUs were mostly affiliated to one of the canonical XoxF clades, i.e., XoxF5 [41]. The underrepresentation of other clades might had been effected by the design strategy of the primers (Fig. S8, Supplemental Information). Only OTU15_mdh_ was closely similar to a *mxaF* gene of *Beijerinckiaceae* (Fig. 3c and Fig. S5). Predominant ^13^C-labeled *mxaF*/*xoxF* OTUs associated with known *xoxF* genes of *Bradyrhizobium* and *Sinorhizobium* were found in all four ^13^C-labeled substrate treatments. On the other hand, ^13^C-labeled *mxaF*/*xoxF* OTUs closely related with *xoxF* genes from *Acetobacteraceae (Acidiphilium, Gammproteobacteria*) were detected in the [^13^C]-CH_3_OH-amended treatment only (OTU14_mdh_) (Figs. 3c and S5). We hypothesize that OTU14_mdh_ corresponds to a methanol utilizer, not a CH_3_Cl utilizer, also because only methanol-utilizing strains are known in *Acetobacteraceae* [43, 44]. In both [^13^C]-CH_3_Cl-amended treatments, OTU25_mdh_ was dominant, whereas in both [^13^C]-CH_3_OH amended treatments, OTU24_mdh_ prevailed. Both OTUs correspond to *Bradyrhizobium*-like *xoxF* genes.

### Key *Bacteria* of the CH_3_Cl sink in the investigated top soil

At first glance, bacterial taxa suggested to be associated with utilization of CH_3_Cl differ depending on which of the three investigated gene biomarkers in the [^13^C]-CH_3_Cl SIP experiment is considered (Fig. 3, Tables 3 and 4). However, consideration of the labeling proportion (LP) of ^13^C-labeled OTUs (Fig. 3) allows to refine the analysis. A high LP suggests strong ^13^C-labeling, hinting at predominant transformation of the ^13^C labeled-substrate by *Alphaproteobacteria*. In support of this, the taxonomic 16S rRNA gene biomarker indeed suggests that this class, and in particular strains closely related to *Methylovirgula* within the *Beijerinckiaceae* family, includes a major part of the primary utilizers of amended [^13^C]-CH_3_Cl soils (Figs. 3a, S4, S6). Beyond that, the LP analysis approach also allowed to identify *Actinobacteria* of the genus *Kineospora* as potential novel key [^13^C]-CH_3_Cl degraders (Figs. 3a, S4, Table S3).

## Discussion

We show in this study that the oxic compartment of top soil was the main sink for CH_3_Cl in the investigated forest soil, and that this degradation process was primarily biotic, in agreement with an early exploratory study of CH_3_Cl dissipation in various environments [27]. We performed a detailed study of potential CH_3_Cl degraders in top soil, and focused on oxic conditions, since anoxic incubations did not show evidence for CH_3_Cl consumption, as expected given that strict anaerobes are not frequently abundant in oxic soils [45].

### Potential bacterial degraders of CH_3_Cl in forest top soil are phylogenetically distinct from known CH_3_Cl-utilizing isolates

*Alphaproteobacteria* (i.e., *Beijerinckiaceae*), and *Actinobacteria* (i.e., *Kineosporaceae*) likely represent key CH_3_Cl degraders in the investigated forest soil (Fig. 3a). The phylogenetic affiliation of these CH_3_Cl utilizers was only distantly related to known CH_3_Cl degraders and thus a novel finding. Moreover, metabolically active *Actinobacteria* show only limited sequence identity with the only known CH_3_Cl-degrading isolate of this phylum, *Nocardioidia* sp. strain SAC-4 [27] (Fig. S4). In contrast, almost all soil CH_3_Cl-degrading isolates known so far are affiliated with only a few genera of *Alphaproteobacteria* [15, 18, 29, 30], and are phylogenetically distinct from the key genus associated with CH_3_Cl degradation in our study (*Methylovirgula*, family *Beijerinckiaceae*). In addition, members of the candidate division *Cand*. Saccharibacteria (syn. TM7) were also ^13^C-labeled in our experiments. Physiological knowledge within this division is limited, and methylotrophy unreported to date [40, 46]. Since cross-labeling via ^13^CO_2_ cannot be fully ruled out in our experimental setup due to the long incubation period, further efforts will be required to confirm that this phylum indeed includes methylotrophs able to degrade CH_3_Cl.

### Low diversity of the *cmuA* biomarker and potential horizontal gene transfer of *cmu* genes to *Beijerinckiaceae*

Amplicons of *cmuA* retrieved from ^13^C-labeled DNA were most closely similar to the *cmuA* gene of *Methylobacterium extorquens* CM4 in both [^13^C]-CH_3_Cl and [^13^C]-CH_3_OH treatments, despite the fact that newly designed *cmuA* primers cover a broader diversity of known *cmuA* sequences than previous ones (Fig. S7). However, abundance of *Methylobacteriaceae* in 16S rRNA gene datasets was <1% (data not shown), and no OTU affiliated to this family was defined as ^13^C-labeled with the applied criteria. Notably, *Beijerinckiaceae* was the key family associated with CH_3_Cl degradation based on the 16S rRNA gene biomarker. This may suggest that CH_3_Cl-degraders from *Beijerinckiaceae* use another CH_3_Cl degradation pathway than the *cmu* pathway, or that they possess a *cmuA* gene that escapes detection with the used primers. Alternatively, such degraders may have acquired a known *cmuA* gene by horizontal gene transfer. Indeed, the *cmu* pathway is plasmid-borne in *M. extorquens* CM4 [47], and further experimental [48] as well as sequence-based [13] evidence for horizontal transfer of the capacity for chloromethane degradation is also available.

### Evidence for novel CH_3_Cl degraders based on *mxaF*/*xoxF*

Whereas *mxaF* genes had been detected in a previous study of the same soil with canonical primers [22] (Fig. S5), mainly *xoxF* sequences were retrieved here with newly designed primers that detect both *mxaF* and *xoxF* (Fig. S5, Supplemental Information). Of the detected 25 *mxaF/xoxF* OTUs, only three OTUs, most similar to *xoxF* genes found in *Bradyrhizobium* and *Sinorhizobium* genomes, dominated microcosms irrespectively of the performed treatment (Fig. 3c). Worthy of note, growth with CH_3_Cl has not been reported to date for representatives of these genera [23, 49, 50]. In addition, *xoxF* genes similar to those of *Methylobacterium* were also ^13^C-labeled upon [^13^C]-CH_3_Cl amendment. Although some cross-feeding of ^13^CO_2_ cannot be entirely excluded since *Bradyrhizobium, Sinorhizobium*, and *Methylobacterium* can assimilate CO_2_ [49, 51], this possibility was minimized by regular exchange of the gas phase to remove formed CO_2_. 16S rRNA gene OTU analysis indicated members of *Beijerinckiceae* as main alphaproteobacterial CH_3_Cl degraders (Fig. 3a). Basing on phylogenetic analysis (Fig. S5), the major ^13^C-labeled *xoxF* OTUs appear quite distinct from known *xoxF* genes of *Beijerinckiceae Sinorhizobium* and *Bradyrhizobium*. Hence, we hypothesize that OTU24_mdh_ and OTU25_mdh_ represent hitherto unknown *xoxF* genes in *Beijerinckiaceae*, or that used primers discriminated against the genotype of *Beijerinckiaceae*. A XoxF-type MDH might be advantageous as it directly leads to formate, avoiding the more toxic formaldehyde produced by MxaFI-type MDH [52].

### Consumption of CH_3_OH by CH_3_Cl degraders and implications for the CH_3_Cl sink in soil

*Beijerinckiaceae* were newly identified here as novel potential CH_3_Cl and also CH_3_OH utilizers. The latter finding agrees well with a previous study on the microbial methanol sink in the same soil [22]. None of the methylotrophic *Beijerinckiaceae* characterized so far utilize methyl halides [53]. *Beijerinckiacea* also comprise typical methanotrophs [54], and one such isolate was shown to transform CH_3_Cl under laboratory conditions, but not to grow with this compound [55, 56]. Basing on the functional gene marker *pmoA* (encoding the beta-subunit of particulate methane monooxygenase), representatives of the uncultivated upland soil cluster α taxon represented the most abundant group of methanotrophs in Steigerwald forest soil. This *pmoA* type, however, is phylogenetically distinct from *pmoA* of *Beijerinckiaceae* [32]. However, a recent study suggest that USCα is indeed a member of *Beijerinckiceae* [57]. Thus, we presently cannot rule out that methanotrophs were not involved in the observed CH_3_Cl sink activity in Steigerwald forest soil.

The presence of CH_3_OH as an alternative methylotrophic growth substrate supports the notion of enhanced assimilation of carbon from CH_3_Cl by *Beijerinckiaceae* during CH_3_OH-driven growth. This idea is supported by the observed high labeling percentages in combined substrate amendments with CH_3_OH and [^13^C]-CH_3_Cl (Fig. 3). Similarly, the reverse combined amendment of unlabeled CH_3_OH with [^13^C]-CH_3_Cl led to increased mineralization of [^13^C]-CH_3_Cl (Fig. S1). Taken together, these findings suggest that activity and growth of soil microorganisms that define the bacterial CH_3_Cl sink in the investigated soil strongly depend on availability of CH_3_OH.

Many methylotrophs can simultaneously utilize several one-carbon compounds [58]. This is likely a selective advantage in natural environments when availability of potential substrates is variable and often limiting. As shown in a previous study on the same soil, *Beijerinckiaceae* can be either methylotrophic or non-methylotrophic [22]. On the basis of the data reported here, we suggest that key CH_3_Cl degraders in soil may be capable of assimilating several one-carbon substrates to optimize their growth. Such a metabolic lifestyle is likely to be of advantage in order to compete with other aerobes in the complex top soil environment, and suggests that the microbial CH_3_Cl sink is linked to the availability of other key carbon sources in soil such as CH_3_OH.

## Conclusions

Our study provides a first deep coverage exploration of bacterial diversity functionally linked with the CH_3_Cl sink in soil. It has revealed that CH_3_Cl consumption in forest soil may be driven by alternative carbon sources such as CH_3_OH. It also uncovered new taxa associated with CH_3_Cl degradation, including genera of *Alphaproteobacteria* and *Actinobacteria* that had not yet been identified in the context of previous SIP experiments with CH_3_Cl, and for which no isolates are yet available. The used CH_3_Cl concentrations, which are much higher than those encountered in the troposphere, might have harmed some bacteria that cannot grow with 1% CH_3_Cl. However, we focused in the study on those soil bacteria that can deal with these concentrations and degraded it. We are aware that the used SIP approach might have been biased by label transfer. However, the low limit of detection of DNA SIP combined with the very conservative approach chosen to identify potential chloromethane degraders, only strongly-labeled microorganisms were identified, which maximizes the likelihood that they were directly labeled from amended labeled chloromethane and not through crossfeeding. Taxa corresponding to cultivated model CH_3_Cl degraders, such as *Methylobacterium extorquens* CM4, were not relevant for CH_3_Cl degradation in the investigated forest top soil. Thus, cultivation of new isolates requires future efforts to improve coverage of existing diversity chloromethane degraders by isolates. Detection of *cmuA* genes closely similar to those of such strains (>99%) suggests that horizontal transfer of the ability to degrade CH_3_Cl is an important aspect of the CH_3_Cl sink in soil. Our study also suggests that methylotrophs in top soil may have a competitive advantage over non-methylotrophs by their ability to utilize diverse one-carbon substrates simultaneously. Testing this hypothesis, and addressing alternative metabolic strategies of CH_3_Cl degradation in soil, will be the topic of future investigations.

## Electronic supplementary material

Supplementa Material
