# Supplementary material for: Methanol consumption drives the bacterial chloromethane sink in a forest soil
Source: ISME J. 2018 Jul 10;12(11):2681–93. doi: 10.1038/s41396-018-0228-4 (PMC6194010; doi:10.1038/s41396-018-0228-4)
Supplement: Supplementary file 1 — Supplementa Material [file 41396_2018_228_MOESM1_ESM.docx]

Contents of the Supplemental Information

Methanol consumption drives the bacterial chloromethane sink in a forest soil

P. Chaignaud, M. Morawe, L. Besaury, E. Kröber, S. Vuilleumier, F. Bringel, S. Kolb

**Supplemental Tables**

**Table S1.** Summary of sequence data (MiSeq, Illumina) obtained in the SIP experiment ………………..…… 2

**Table S2**. Newly designed and used primers for PCR targeting *cmuA* and *mxaF*/*xoxF* genes ……………….. **3**

**Table S3.** Overview of phylogenetic affiliation, relative abundance, and ^13^C-label of all detected phylotypes of analysed gene biomarkers of the SIP experiment …………………………………………………………...….. 4

**Supplemental Figures**

**Figure S1.** CO_2_ formation during the SIP experiment …… ………………………………………………….. 15

**Figure S2.** CsCl buoyant density of gradient fractions (a) and relative distribution of DNA (b) …………….. 16

**Figure S3.** NMDS analyses of the 16S rRNA gene (a), *cmuA* (b) and *mxaF/xoxF* phylotypes (c) from the SIP experiment …………………………………………………………………………………………………….. 17

**Figure S4.** Phylogenetic tree of all labelled bacterial phylotypes (16S rRNA gene) …………………...…….. 18

**Figure S5.** Phylogenetic tree all *mxaF* and *xoxF* phylotypes obtained ……………………………………….. 19

**Figure S6.** Relative abundances of bacterial 16S rRNA phylotypes ………………………………………….. 23

**Supplemental Information on Materials & Methods**

**Figure S7**. Comparing two primers sets targeting *cmuA* ……………………………………………………… 24

**Figure S8.** Rationale for new primer design targeting PCR amplification of methanol deshydrogenase (mdh) encoding *mxaF* / *xoxF* genes …………………………………………...………………………………...…… 25

**List 1.** CmuA-like nucleotide database entries used to design the new primers cmuAf422 and cmuAr422 ….. 28

**List 2.** MxaF/XoxF nucleotide database entries used to design the new primers mdh1/mdh2 and mdhR …… 30

**References Lists of Supplemental Information** ……………………………………………………. 33

| **Microcosm** | **DNA fraction^a^** | **Total filtered reads^b^16S rRNA** | **Filtered reads^b^ 16S rRNA** |  | **Total filtered reads^b^** | |  | **Filtered reads** | | |
| --- | --- | --- | --- | --- | --- | --- | --- | --- | --- | --- |
|  |  |  |  |  | ***cmuA*** | ***mxaF/xoxF*** |  | ***cmuA*** | ***mxaF/xoxF*** |  |
| [^13^C]-CH_3_Cl | H | 205130 | 46110 |  | 5382 | 2283 |  | 4282 | 786 |  |
|  | L |  | 152348 |  |  |  |  | 1100 | 1452 |  |
| [^13^C]-CH_3_Cl + CH_3_OH | H | 235958 | 178042 |  | 5936 | 3102 |  | 3778 | 1534 |  |
|  | L |  | 50402 |  |  |  |  | 2158 | 1568 |  |
| CH_3_Cl +  [^13^C]-CH_3_OH | H | 189556 | 76690 |  | 5864 | 2406 |  | 614 | 918 |  |
|  | L |  | 106222 |  |  |  |  | 5250 | 1488 |  |
| [^13^C]-CH_3_OH | H | 139666 | 98332 |  | 2760 | 1776 |  | 1886 | 588 |  |
|  | L |  | 37886 |  |  |  |  | 874 | 1188 |  |
| CH_3_Cl | H | 149230 | 72522 |  | 3450 | 4476 |  | 370 | 2616 |  |
|  | L |  | 71882 |  |  |  |  | 3080 | 1860 |  |
| CH_3_OH + CH_3_Cl | H | 142956 | 47236 |  | 4294 | 2544 |  | 922 | 862 |  |
|  | L |  | 91184 |  |  |  |  | 3372 | 1682 |  |
| CH_3_OH | H | 20302 | 90822 |  | 2706 | 4012 |  | 246 | 2946 |  |
|  | L |  | 103596 |  |  |  |  | 2460 | 1066 |  |
| Control  (without substrate supplementation) | H | 165754 | 100032 |  | 3814 | 4382 |  | 184 | 2710 |  |
|  | L |  | 60798 |  |  |  |  | 3630 | 1672 |  |

**Table S1. Summary of sequence data (MiSeq, Illumina) obtained in the SIP experiment**

^a^ H, L: heavy and light DNA fractions respectively; ^b^ Sum of filtered reads in fractions H and L for each microcosm; see Materials and Methods for further details.

Table S2. Newly designed and used primers for PCR targeting *cmuA* and *mxaF*/*xoxF* genes

| **gene** | **Product size** | **Primer orientation, name, sequence 5'->3‘ (degeneracy rate)^a^** | **CM4 genome position (nt)^b^** | **GC content (%)** | **Taxonomical affiliation of mismatching OTU sequences for *N* aligned sequences (number of sequences) ^c^** | **Reference** |
| --- | --- | --- | --- | --- | --- | --- |
| *cmuA* | 442bp | Forward, cmuA802F, TTCAACGGCGAYATGTATCCYGG (4) | 336191-336213 | 52.2 | *Thermosediminibacter ocean*i*, Desulfotomaculum alcoholivorax* (31) | (Miller *et al.*, 2004) |
|  |  | Reverse, cmuA1244R, TABTCCATDATGGCYTCGAC (18) | 335790-335771 | 56.3 | Marine methyl halide utilising bacteria, *Thermosediminibacter ocean*i, *Thermincola potens*, *Desulfomonile* *tiedjei*, *Desulfotomaculum alcoholivorax* (111) | (Farhan Ul Haque *et al.*, 2017) |
|  | 422bp (new) | Forward, cmuAf422 GARGTBGGITAYAAYGGHGG (24) | 336046-336066 | 52.5 | (111) | This work |
|  |  | Reverse, cmuAr422 TCRTTGCGCTCRTACATGTCICC (4) | 335666-335644 | 52.2 | *Thermosediminibacter ocean*i, *Desulfomonile* *tiedjei*, uncultured bacterium (111) | This work |
| *mxaF/xoxF* | 549bp | Forward, mxaF_1003f GCGGCACCAACTGGGGCTCGT | 4786435-786452 | 67.6 | *Hyhomicrobium denitrificans* ATCC 51888, *Hyphomicrobium sp*. MC1, *Hyphomicrobium nitrativorans* NL23, *Methylocella silvestris* BL2, *Methylobacterium extorquens* PA1, *Methylobacillus flagellatus* KT, *Methylobacterium nodulans* strain ORS2060 (nd) | (McDonald and Murrell, 1997) |
|  |  | Reverse, mxaF_1555r CATGAABGGCTCCCARTCCAT (6) | 4785922-785902 | 59.5 | nd | (Neufeld *et al.*, 2007a) |
|  | 551bp(new) | Forward, mdh1 and mdh2 GCGGIWSCAICTGGGGYT (8) GCGGIWSGAICTGGGGYT (8) | 4786432-786452 4786432-786452 | 69.0 | *Methylocella silvestris* BL2 (120) | This work |
|  |  | Reverse, mdhR GAASGGYTCSYARTCCATGCA (32) | 4785925-785905 | 55.5 | (120) | This work |

**^a^** Number of different oligomers per synthesized degenerate primer with B=C/G/T, D=A/G/T, H=A/C/T, I=inosine, R=A/G, S=G/C, Y=C/T, V=A/C/G, W=A/T;

**^b^** MaGe database <http://www.genoscope.cns.fr/agc/mage>);  **^c^** Divergent sequences include sequences with a 3'-terminal mismatch, or one mismatch at the second last position of the 3'-terminal position or with three or more mismatches. Divergent gene *cmuA* sequence accession n° Desti_5447 (*Desulfomonile* *tiedjei* DSM 6799, Uniprot); TherJR_0143 (*Thermincola potens* JR, Uniprot); Toce_1533 (*Thermosediminibacter ocean*i DSM 16646, Uniprot); WP_027363948 (*Desulfotomaculum* *alcoholivorax*, GenBank); Gi/66474906 (Marine methyl halide utilizing bacteria); Gi/664795/1 (Uncultured bacteria). Divergent *mxaF­*/*xoxF* sequences include sequences accession n° Hden_1305/ Hden_1617/ Hden_2848 (*Hyhomicrobium denitrificans* ATCC 51888, Uniprot); HYPMCv2_0800/ HYPMCv2_3613 (*Hyphomicrobium* sp. MC1, Uniprot); Mfla_1451 (*Methylobacillus flagellatus* KT, Uniprot); Mext_0099 (*Methylobacterium extorquens* PA1); Mnod_2344 (*Methylobacterium nodulans* strain ORS2060, Uniprot); Msil_2260/ Q8KMI4_METSI (*Methylocella silvestris* BL2, Uniprot); W911_13290 (*Hyphomicrobium nitrativorans* NL23, Uniprot)

**^c^** Divergent sequences include sequences either with one mismatch at the second last position of the 3'-terminal or with more than three mismatches.

**Table S3. Overview of phylogenetic affiliation, relative abundance, and ^13^C-label of all detected phylotypes of analysed gene biomarkers of the SIP experiment.**

|  |  | |  | | | **treatment^a .^** | | **^12^CH_3_OH** | |  | **^13^CH_3_OH** | |  | **^12^CH_3_Cl** | |  | **^13^CH_3_Cl** | |  | **^12^CH_3_OH &  ^12^CH_3_Cl** | |  | **^12^CH_3_OH &  ^12^CH_3_Cl** | |  |  | **^12^CH_3_OH &  ^13^CH_3_Cl** | | |  |
| --- | --- | --- | --- | --- | --- | --- | --- | --- | --- | --- | --- | --- | --- | --- | --- | --- | --- | --- | --- | --- | --- | --- | --- | --- | --- | --- | --- | --- | --- | --- |
|  | **phylogenetic affiliation^b^** | | | | | | | **%-abundance in fraction^c^** | | | | | **^13^C-label^d^** | **%-abundance in fraction^c^** | | | | | **^13^C-label^d^** | **%-abundance in fraction^c^** | | | | | **^13^C-label^d^** |  | **%-ab. fract. ^c^** | | | **^13^C-label^d^** |
| **OTU** | ***phylum* / *class* or  environmental group** | | | ***order*** | | ***family*  *(genus)*** | | **H** | **L** |  | **H** | **L** |  | **H** | **L** |  | **H** | **L** |  | **H** | **L** |  | **H** | **L** |  |  | **H** | **L** | |  |
| ***16S rRNA* phylotypes** | | | | | | | | | | | | | | | | | | | | | | | | | | | | | | |
| OTU1_16S_ | | A21b | | n.a. | | n.a. | | 0.02 | - |  | - | - |  | 0.03 | - |  | - | 0.01 |  | 0.02 | - |  | 0.01 | 0.01 |  |  | - | - |  | |
| OTU2_16S_ | | ABS-6 | | n.a. | | n.a. | | 0.03 | - |  | 0.01 | - |  | 0.02 | 0.02 |  | 0.02 | 0.02 |  | 0.03 | 0.03 |  | 0.01 | 0.04 |  |  | 0.02 | - |  | |
| OTU3_16S_ | | *Alphaproteobacteria* | | *Rhodospirillales* | | *Acetobacteraceae* | | 2.50 | 1.53 |  | 1.33 | 1.70 |  | 2.56 | 1.55 |  | 1.69 | 1.80 |  | 2.20 | 1.81 |  | 1.55 | 1.36 |  |  | 1.91 | 1.45 |  | |
| OTU4_16S_ | | *Actinobacteria* | | *Acidimicrobiales* | | n.a. | | 0.09 | 0.02 |  | - | 0.02 |  | 0.05 | 0.05 |  | 0.04 | 0.03 |  | 0.05 | 0.03 |  | 0.01 | 0.02 |  |  | 0.02 | 0.02 |  | |
| OTU5_16S_ | | *Acidobacteria* | | *Acidobacteriales* | | *Acidobacteriaceae* | | 11.12 | 17.49 |  | 4.05 | 13.23 |  | 6.67 | 11.44 |  | 3.17 | 8.87 |  | 5.28 | 8.11 |  | 3.15 | 8.76 |  |  | 4.22 | 8.09 |  | |
| OTU6_16S_ | | *Actinobacteria* | | *Actinomycetales* | | n.a. | | 5.33 | 2.22 |  | 2.82 | 3.27 |  | 7.38 | 3.79 |  | 12.91 | 5.12 | **+** | 5.89 | 4.96 |  | 6.01 | 5.04 |  |  | 7.29 | 4.57 |  | |
| OTU7_16S_ | | AK1AB1 | | n.a. | | n.a. | | 0.11 | 0.09 |  | 0.03 | 0.17 |  | 0.15 | 0.07 |  | 0.14 | 0.23 |  | 0.08 | 0.26 |  | 0.05 | 0.19 |  |  | 0.11 | 0.24 |  | |
| OTU8_16S_ | | n.a. | | n.a. | | *Anaerobrancaceae* | | - | - |  | 0.01 | - |  | 0.02 | - |  | - | - |  | - | - |  | 0.01 | 0.01 |  |  | - | - |  | |
| OTU9_16S_ | | At12OctB3 | | n.a. | | n.a. | | - | 0.01 |  | - | - |  | - | - |  | - | - |  | - | - |  | - | 0.01 |  |  | - | - |  | |
| OTU10_16S_ | | auto67 | | n.a. | | n.a. | | 0.64 | 0.54 |  | 0.23 | 0.17 |  | 0.14 | 0.06 |  | 0.14 | 0.09 |  | 0.12 | 0.05 |  | 0.02 | 0.08 |  |  | 0.09 | 0.03 |  | |
| OTU11_16S_ | | *Firmicutes* | | *Bacillales* | | *Bacillaceae* | | 0.77 | 0.57 |  | 0.27 | 0.50 |  | 0.84 | 0.78 |  | 0.68 | 0.75 |  | 2.15 | 0.61 |  | 0.31 | 0.85 |  |  | 0.93 | 0.63 |  | |
| OTU12_16S_ | | *Alphaproteobacteria* | | *Rhizobiales* | | *Beijerinckiaceae* | | 14.24 | 16.20 |  | 59.35 | 18.22 | **+** | 16.93 | 20.20 |  | 27.93 | 17.57 | **+** | 17.39 | 21.35 |  | 13.52 | 18.96 |  |  | 31.15 | 14.77 | **+** | |
| OTU13_16S_ | | *Alphaproteobacteria* | | *Rhizobiales* | | *Bradyrhizobiaceae* | | 0.28 | 0.22 |  | 0.06 | 0.23 |  | 0.12 | 0.27 |  | 0.16 | 0.24 |  | 0.20 | 0.24 |  | 0.13 | 0.11 |  |  | 0.12 | 0.08 |  | |
|  | |  |  | | | **treatment^a .^** | | **^12^CH_3_OH** | |  | **^13^CH_3_OH** | |  | **^12^CH_3_Cl** | |  | **^13^CH_3_Cl** | |  | **^12^CH_3_OH &  ^12^CH_3_Cl** | |  | **^12^CH_3_OH &  ^12^CH_3_Cl** | |  |  | **^12^CH_3_OH &  ^13^CH_3_Cl** | | |  |
|  | | **phylogenetic affiliation^b^** | | | | | | **%-abundance in fraction^c^** | | | | | **^13^C-label^d^** | **%-abundance in fraction^c^** | | | | | **^13^C-label^d^** | **%-abundance in fraction^c^** | | | | | **^13^C-label^d^** |  | **%-ab. fract. ^c^** | | | **^13^C-label^d^** |
| **OTU** | | ***phylum* / *class* or  environmental group** | | ***order*** | | ***family*  *(genus)*** | | **H** | **L** |  | **H** | **L** |  | **H** | **L** |  | **H** | **L** |  | **H** | **L** |  | **H** | **L** |  |  | **H** | **L** | |  |
| OTU15_16S_ | | *Betaproteobacteria* | | *Burlholderiales* | | *Burkholderiaceae* | | 0.23 | 0.14 |  | 0.17 | 0.10 |  | 0.20 | 0.10 |  | 0.07 | 0.13 |  | 0.22 | 0.15 |  | 0.10 | 0.16 |  |  | 0.16 | 0.12 |  | |
| OTU16_16S_ | | C111 | | n.a. | | n.a. | | - | - |  | - | - |  | - | - |  | - | - |  | - | - |  | - | - |  |  | - | - |  | |
| OTU17_16S_ | | *Alphaproteobacteria* | | *Caulobacterales* | | *Caulobacteraceae* | | 0.45 | 0.22 |  | 0.18 | 0.25 |  | 0.29 | 0.25 |  | 0.30 | 0.14 |  | 0.40 | 0.23 |  | 0.06 | 0.25 |  |  | 0.21 | 0.08 |  | |
| OTU18_16S_ | | *Bacteroidetes* | | *Sphingobacteriales* | | *Chitinophagaceae* | | 0.62 | 0.36 |  | 0.27 | 0.46 |  | 0.13 | 0.10 |  | 0.04 | 0.06 |  | 0.15 | 0.06 |  | 0.05 | 0.08 |  |  | 0.10 | 0.03 |  | |
| OTU19_16S_ | | *Gammaproteobacteria* | | *Chromatiales* | | *Chromatiaceae* | | 1.53 | 2.11 |  | 0.79 | 2.10 |  | 0.81 | 1.58 |  | 0.52 | 1.40 |  | 1.51 | 1.30 |  | 0.55 | 1.51 |  |  | 0.65 | 1.67 |  | |
| OTU20_16S_ | | *Verrucomicrobia* | | *Chthoniobacterales* | | *Chthoniobacteraceae* | | 0.17 | 0.14 |  | 0.07 | 0.10 |  | 0.15 | 0.05 |  | 0.05 | 0.16 |  | 0.17 | 0.03 |  | 0.11 | 0.08 |  |  | 0.13 | 0.12 |  | |
| OTU21_16S_ | | *Firmicutes* | | *Clostridiales* | | *Clostridiaceae* | | - | - |  | 0.01 | 0.02 |  | 0.01 | - |  | - | - |  | 0.02 | - |  | - | - |  |  | - | - |  | |
| OTU22_16S_ | | *Betaproteobacteria* | | *Burkholderiales* | | *Comamonadaceae* | | 0.08 | 0.13 |  | 0.04 | 0.13 |  | 0.07 | 0.06 |  | 0.07 | 0.07 |  | 0.03 | 0.08 |  | 0.07 | 0.07 |  |  | 0.08 | 0.10 |  | |
| OTU23_16S_ | | *Actinobacteria* | | *Solirubrobacterales* | | *Conexibacteraceae* | | 1.73 | 1.00 |  | 1.09 | 1.30 |  | 3.17 | 2.04 |  | 2.43 | 2.89 |  | 3.40 | 3.14 |  | 1.52 | 2.68 |  |  | 2.43 | 2.79 |  | |
| OTU24_16S_ | | *Actinobacteria* | | *Actinomycetales* | | *Corynebacteriaceae* | | 0.03 | 0.02 |  | 0.01 | - |  | - | 0.02 |  | 0.05 | 0.03 |  | 0.17 | 0.02 |  | 0.01 | 0.02 |  |  | 0.01 | 0.02 |  | |
| OTU25_16S_ | | *Gammaproteobacteria* | | *Legionellales* | | *Coxiellaceae* | | - | - |  | - | - |  | - | 0.01 |  | 0.02 | - |  | - | 0.01 |  | - | - |  |  | 0.01 | - |  | |
| OTU26_16S_ | | *Bacteroidetes* | | *Sphingobacteriales* | | *Cytophagaceae* | | 1.18 | 0.20 |  | 0.36 | 0.15 |  | 0.01 | - |  | - | - |  | - | - |  | - | - |  |  | 0.00 | - |  | |
| OTU27_16S_ | | *Actinobacteria* | | *Actinomycetales* | | *Dietziaceae* | | - | - |  | - | 0.02 |  | - | - |  | - | 0.01 |  | - | 0.01 |  | - | 0.01 |  |  | 0.01 | - |  | |
| OTU28_16S_ | | EB1017 | | n.a. | | n.a. | | 0.06 | 0.06 |  | 0.04 | 0.08 |  | 0.07 | 0.07 |  | 0.09 | 0.03 |  | 0.13 | 0.06 |  | 0.01 | 0.03 |  |  | 0.06 | 0.02 |  | |
| OTU29_16S_ | | Ellin329 | | n.a. | | n.a. | | - | - |  | - | - |  | - | - |  | - | - |  | - | - |  | 0.01 | - |  |  | - | - |  | |
|  | |  |  | | | **treatment^a .^** | | **^12^CH_3_OH** | |  | **^13^CH_3_OH** | |  | **^12^CH_3_Cl** | |  | **^13^CH_3_Cl** | |  | **^12^CH_3_OH &  ^12^CH_3_Cl** | |  | **^12^CH_3_OH &  ^12^CH_3_Cl** | |  |  | **^12^CH_3_OH &  ^13^CH_3_Cl** | | |  |
|  | | **phylogenetic affiliation^b^** | | | | | | **%-abundance in fraction^c^** | | | | | **^13^C-label^d^** | **%-abundance in fraction^c^** | | | | | **^13^C-label^d^** | **%-abundance in fraction^c^** | | | | | **^13^C-label^d^** |  | **%-ab. fract. ^c^** | | | **^13^C-label^d^** |
| **OTU** | | ***phylum* / *class* or  environmental group** | | ***order*** | | ***family*  *(genus)*** | | **H** | **L** |  | **H** | **L** |  | **H** | **L** |  | **H** | **L** |  | **H** | **L** |  | **H** | **L** |  |  | **H** | **L** | |  |
| OTU30_16S_ | | Ellin515 | | n.a. | | n.a. | | 0.01 | - |  | - | - |  | 0.01 | 0.01 |  | - | - |  | - | - |  | - | - |  |  | 0.01 | - |  | |
| OTU31_16S_ | | Ellin5290 | | n.a. | | n.a. | | 0.02 | 0.02 |  | 0.02 | 0.02 |  | 0.03 | 0.02 |  | 0.04 | 0.03 |  | 0.03 | 0.01 |  | - | 0.02 |  |  | 0.03 | 0.03 |  | |
| OTU32_16S_ | | Ellin6513 | | n.a. | | n.a. | | 0.54 | 0.49 |  | 0.35 | 0.52 |  | 0.39 | 0.36 |  | 0.36 | 0.26 |  | 0.49 | 0.29 |  | 0.20 | 0.30 |  |  | 0.34 | 0.15 |  | |
| OTU33_16S_ | | *Gammaproteobacteria* | | *Enterobacteriales* | | *Enterobacteriaceae* | | - | - |  | 0.03 | - |  | 0.01 | 0.02 |  | 0.50 | - |  | 5.80 | 0.07 |  | - | - |  |  | 0.02 | 0.02 |  | |
| OTU34_16S_ | | *Firmicutes* | | *Clostridiales* | | *Eubacteriaceae* | | - | - |  | 0.01 | - |  | - | - |  | - | - |  | - | - |  | - | - |  |  | 0.00 | - |  | |
| OTU35_16S_ | | FBP | | n.a. | | n.a. | | - | - |  | 0.01 | - |  | - | - |  | - | - |  | - | - |  | - | - |  |  | - | - |  | |
| OTU36_16S_ | | *Armatimonadetes* | | *Fimbriimonadales* | | n.a. | | - | - |  | - | - |  | - | 0.01 |  | - | - |  | - | - |  | - | - |  |  | 0.00 | - |  | |
| OTU37_16S_ | | *Bacteroidetes* | | *Flavobacteriales* | | *Flavobacteriaceae* | | 0.55 | 0.40 |  | 0.21 | 0.27 |  | 0.24 | 0.42 |  | 0.12 | 0.44 |  | 0.37 | 0.19 |  | 0.07 | 0.43 |  |  | 0.25 | 0.20 |  | |
| OTU38_16S_ | | *Actinobacteria* | | *Frankiales* | | *Frankiaceae* | | 0.02 | - |  | - | - |  | 0.03 | - |  | - | 0.01 |  | 0.07 | 0.01 |  | - | 0.02 |  |  | 0.01 | 0.02 |  | |
| OTU39_16S_ | | *Actinobacteria* | | *Gaiellales* | | *Gaiellaceae* | | 0.17 | 0.15 |  | 0.12 | 0.19 |  | 0.55 | 0.23 |  | 0.34 | 0.29 |  | 0.40 | 0.27 |  | 0.27 | 0.30 |  |  | 0.24 | 0.46 |  | |
| OTU40_16S_ | | GAL15 | | n.a. | | n.a. | | - | - |  | - | - |  | - | - |  | - | - |  | 0.02 | - |  | - | - |  |  | - | - |  | |
| OTU41_16S_ | | Gemm-1 | | n.a. | | n.a. | | - | - |  | - | - |  | - | - |  | - | - |  | 0.02 | - |  | - | - |  |  | - | - |  | |
| OTU42_16S_ | | *Planctomycetes* | | *Planctomycetales* | | *Gemmataceae* | | 2.76 | 2.31 |  | 1.33 | 2.29 |  | 2.20 | 1.93 |  | 0.75 | 1.78 |  | 1.98 | 1.50 |  | 0.64 | 1.50 |  |  | 1.27 | 1.88 |  | |
| OTU43_16S_ | | *Gemmatimonadetes* | | *Gemmatimonadeles* | | n.a. | | - | - |  | - | - |  | - | - |  | - | 0.01 |  | 0.03 | - |  | 0.01 | - |  |  | - | - |  | |
| OTU44_16S_ | | *Actinobacteria* | | *Geodermatophilales* | | *Geodermatophilaceae* | | 0.05 | 0.06 |  | 0.09 | 0.08 |  | 0.23 | 0.11 |  | 0.09 | 0.14 |  | 0.20 | 0.11 |  | 0.23 | 0.20 |  |  | 0.46 | 0.05 |  | |
|  | |  |  | | | **treatment^a .^** | | **^12^CH_3_OH** | |  | **^13^CH_3_OH** | |  | **^12^CH_3_Cl** | |  | **^13^CH_3_Cl** | |  | **^12^CH_3_OH &  ^12^CH_3_Cl** | |  | **^12^CH_3_OH &  ^12^CH_3_Cl** | |  |  | **^12^CH_3_OH &  ^13^CH_3_Cl** | | |  |
|  | | **phylogenetic affiliation^b^** | | | | | | **%-abundance in fraction^c^** | | | | | **^13^C-label^d^** | **%-abundance in fraction^c^** | | | | | **^13^C-label^d^** | **%-abundance in fraction^c^** | | | | | **^13^C-label^d^** |  | **%-ab. fract. ^c^** | | | **^13^C-label^d^** |
| **OTU** | | ***phylum* / *class* or  environmental group** | | ***order*** | | ***family*  *(genus)*** | | **H** | **L** |  | **H** | **L** |  | **H** | **L** |  | **H** | **L** |  | **H** | **L** |  | **H** | **L** |  |  | **H** | **L** | |  |
| OTU46_16S_ | | *Deltaproteobacteria* | | *Myxococcales* | | *Haliangiaceae* | | - | 0.01 |  | 0.03 | 0.02 |  | - | - |  | - | 0.01 |  | 0.03 | 0.02 |  | 0.01 | - |  |  | 0.01 | 0.02 |  | |
| OTU47_16S_ | | *Gammaproteobacteria* | | *Oceanospirillales* | | *Halomonadaceae* | | - | 0.01 |  | - | 0.06 |  | - | 0.02 |  | - | 0.01 |  | 0.07 | 0.04 |  | - | 0.02 |  |  | - | - |  | |
| OTU49_16S_ | | *Alphaproteobacteria* | | *Rhizobiales* | | *Hyphomicrobiaceae* | | 2.64 | 4.51 |  | 2.44 | 4.70 |  | 2.28 | 4.55 |  | 2.41 | 3.83 |  | 2.82 | 4.20 |  | 1.86 | 4.26 |  |  | 2.18 | 3.52 |  | |
| OTU51_16S_ | | *Actinobacteria* | | *Micrococcales* | | *Intrasporangiaceae* | | - | 0.01 |  | 0.02 | 0.02 |  | - | 0.01 |  | - | - |  | 0.02 | 0.01 |  | 0.01 | 0.01 |  |  | 0.01 | - |  | |
| OTU52_16S_ | | IS-44 | | n.a. | | n.a. | | - | - |  | - | - |  | - | - |  | 0.02 | - |  | - | - |  | - | - |  |  | - | - |  | |
| OTU53_16S_ | | *Planctomycetes* | | *Planctomycetales* | | *Isosphaeraceae* | | 11.80 | 13.58 |  | 5.36 | 15.29 |  | 10.28 | 9.88 |  | 3.34 | 7.56 |  | 6.71 | 8.26 |  | 3.51 | 9.41 |  |  | 4.48 | 11.52 |  | |
| OTU54_16S_ | | n.a. | | n.a. | | *Coriobacteriaceae* | | - | - |  | - | - |  | 0.01 | - |  | - | - |  | - | - |  | - | - |  |  | - | - |  | |
| OTU55_16S_ | | *Firmicutes* | | *Clostridiales* | | *Lachnospiraceae* | | - | - |  | - | - |  | 0.01 | - |  | - | - |  | 0.02 | - |  | - | - |  |  | - | - |  | |
| OTU56_16S_^e^ | | plants | | n.a. | | *Lamiaceae* | | 0.08 | 0.02 |  | 0.01 | - |  | 0.07 | 0.01 |  | 0.05 | 0.02 |  | 0.07 | 0.03 |  | 0.01 | 0.02 |  |  | 0.05 | 0.02 |  | |
| OTU57_16S_ | | *Gammaproteobacteria* | | *Legionellales* | | n.a. | | 0.04 | 0.02 |  | 0.01 | 0.02 |  | 0.02 | - |  | 0.02 | 0.01 |  | - | 0.01 |  | - | 0.01 |  |  | 0.02 | - |  | |
| OTU58_16S_ | | *Verrucomicrobia* | | *Methylacidiphilales* | | n.a. | | - | - |  | 0.01 | - |  | - | - |  | - | 0.01 |  | - | - |  | - | - |  |  | - | - |  | |
| OTU59_16S_ | | *Alphaproteobacteria* | | *Rhizobiales* | | *Methylobacteriaceae* | | 0.01 | 0.04 |  | 0.01 | 0.04 |  | 0.02 | 0.04 |  | - | 0.02 |  | - | 0.04 |  | - | 0.01 |  |  | 0.01 | 0.07 |  | |
| OTU60_16S_ | | *Alphaproteobacteria* | | *Rhizobiales* | | *Methylocystaceae* | | 1.02 | 0.76 |  | 0.80 | 0.90 |  | 1.23 | 0.98 |  | 0.68 | 1.11 |  | 0.84 | 1.02 |  | 0.68 | 0.70 |  |  | 0.80 | 0.59 |  | |
| OTU61_16S_ | | *Actinobacteria* | | *Micrococcales* | | *Microbacteriaceae* | | 0.27 | 0.27 |  | 0.15 | 0.44 |  | 0.37 | 0.41 |  | 0.34 | 0.61 |  | 0.52 | 0.57 |  | 30.01 | 0.64 | **+** |  | 0.57 | 0.69 |  | |
| OTU62_16S_ | | *Actinobacteria* | | *Micrococcales* | | *Micrococcaceae* | | 0.02 | - |  | 0.01 | - |  | - | - |  | 0.02 | - |  | - | - |  | - | - |  |  | 0.00 | - |  | |
|  | |  |  | | | **treatment^a .^** | | **^12^CH_3_OH** | |  | **^13^CH_3_OH** | |  | **^12^CH_3_Cl** | |  | **^13^CH_3_Cl** | |  | **^12^CH_3_OH &  ^12^CH_3_Cl** | |  | **^12^CH_3_OH &  ^12^CH_3_Cl** | |  |  | **^12^CH_3_OH &  ^13^CH_3_Cl** | | |  |
|  | | **phylogenetic affiliation^b^** | | | | | | **%-abundance in fraction^c^** | | | | | **^13^C-label^d^** | **%-abundance in fraction^c^** | | | | | **^13^C-label^d^** | **%-abundance in fraction^c^** | | | | | **^13^C-label^d^** |  | **%-ab. fract. ^c^** | | | **^13^C-label^d^** |
| **OTU** | | ***phylum* / *class* or  environmental group** | | ***order*** | | ***family*  *(genus)*** | | **H** | **L** |  | **H** | **L** |  | **H** | **L** |  | **H** | **L** |  | **H** | **L** |  | **H** | **L** |  |  | **H** | **L** | |  |
| OTU63_16S_ | | *Actinobacteria* | | *Actinomycetales* | | *Micromonosporaceae* | | 0.11 | 0.04 |  | 0.12 | 0.10 |  | 0.14 | 0.08 |  | 0.14 | 0.10 |  | 0.17 | 0.10 |  | 0.06 | 0.06 |  |  | 0.14 | 0.12 |  | |
| OTU64_16S_ | | ML635J-21 | | n.a. | | n.a. | | 0.02 | 0.01 |  | - | - |  | - | - |  | - | 0.01 |  | - | 0.01 |  | - | 0.01 |  |  | 0.00 | 0.02 |  | |
| OTU65_16S_ | | *Gammaproteobacteria* | | *Pseudomonadales* | | *Moraxellaceae* | | 0.02 | 0.03 |  | 0.02 | 0.08 |  | 0.01 | 0.05 |  | 0.02 | 0.03 |  | 0.18 | 0.04 |  | 0.02 | 0.05 |  |  | 0.02 | 0.08 |  | |
| OTU66_16S_ | | *Actinobacteria* | | *Actinomycetales* | | *Mycobacteriaceae* | | 0.11 | 0.03 |  | 0.06 | - |  | 0.16 | 0.04 |  | 0.07 | 0.05 |  | 0.13 | 0.04 |  | 0.14 | 0.04 |  |  | 0.08 | 0.05 |  | |
| OTU67_16S_ | | *Deltaproteobacteria* | | *Myxococcales* | | n.a. | | 0.02 | 0.03 |  | 0.01 | - |  | 0.04 | 0.05 |  | - | 0.03 |  | 0.05 | 0.04 |  | 0.01 | 0.03 |  |  | 0.03 | 0.02 |  | |
| OTU68_16S_ | | N1423WL | | n.a. | | n.a. | | - | - |  | - | - |  | - | 0.01 |  | - | - |  | - | - |  | - | - |  |  | - | - |  | |
| OTU69_16S_ | | *Actinobacteria* | | *Nakamurellales* | | *Nakamurellaceae* | | 0.10 | 0.02 |  | 0.06 | 0.04 |  | 0.22 | 0.14 |  | 0.23 | 0.14 |  | 0.29 | 0.17 |  | 0.12 | 0.10 |  |  | 0.18 | 0.10 |  | |
| OTU70_16S_ | | *Betaproteobacteria* | | *Neisseriales* | | *Neisseriaceae* | | 0.06 | 0.18 |  | 0.01 | 0.19 |  | - | 0.12 |  | 0.07 | 0.14 |  | 0.10 | 0.09 |  | 0.06 | 0.06 |  |  | 0.03 | 0.19 |  | |
| OTU71_16S_ | | *Actinobacteria* | | *Nitriliruptorales* | | *Nitriliruptoraceae* | | 0.01 | - |  | - | - |  | - | - |  | - | - |  | - | 0.01 |  | - | 0.02 |  |  | - | - |  | |
| OTU72_16S_ | | *Cyanobacteria* | | *Nostocophycideae* | | n.a. | | 0.02 | - |  | 0.01 | - |  | - | - |  | - | - |  | 0.02 | - |  | - | - |  |  | 0.00 | - |  | |
| OTU73_16S_ | | OC31 | | n.a. | | n.a. | | 0.04 | 0.11 |  | 0.02 | 0.08 |  | 0.04 | 0.04 |  | - | 0.03 |  | 0.02 | 0.04 |  | 0.01 | 0.02 |  |  | 0.04 | 0.07 |  | |
| OTU74_16S_ | | OM27 | | n.a. | | n.a. | | 0.01 | - |  | - | - |  | - | 0.01 |  | - | - |  | - | 0.01 |  | 0.01 | - |  |  | 0.00 | - |  | |
| OTU75_16S_ | | *Verrucomicrobia* | | *Opitutales* | | *Opitutaceae* | | 0.02 | 0.01 |  | - | - |  | - | 0.01 |  | - | 0.01 |  | 0.02 | - |  | - | 0.01 |  |  | 0.00 | 0.02 |  | |
| OTU76_16S_ | | *Betaproteobacteria* | | *Burkholderiales* | | *Oxalobacteraceae* | | 0.13 | 0.14 |  | 0.03 | 0.06 |  | 0.01 | 0.02 |  | - | 0.02 |  | - | 0.03 |  | 0.02 | - |  |  | 0.01 | 0.05 |  | |
| OTU77_16S_ | | *Firmicutes* | | *Bacillales* | | *Paenibacillaceae* | | 0.02 | 0.01 |  | 0.01 | - |  | 0.04 | 0.04 |  | - | 0.03 |  | - | 0.03 |  | 0.02 | 0.01 |  |  | 0.06 | 0.03 |  | |
|  | |  |  | | | **treatment^a .^** | | **^12^CH_3_OH** | |  | **^13^CH_3_OH** | |  | **^12^CH_3_Cl** | |  | **^13^CH_3_Cl** | |  | **^12^CH_3_OH &  ^12^CH_3_Cl** | |  | **^12^CH_3_OH &  ^12^CH_3_Cl** | |  |  | **^12^CH_3_OH &  ^13^CH_3_Cl** | | |  |
|  | | **phylogenetic affiliation^b^** | | | | | | **%-abundance in fraction^c^** | | | | | **^13^C-label^d^** | **%-abundance in fraction^c^** | | | | | **^13^C-label^d^** | **%-abundance in fraction^c^** | | | | | **^13^C-label^d^** |  | **%-ab. fract. ^c^** | | | **^13^C-label^d^** |
| **OTU** | | ***phylum* / *class* or  environmental group** | | | ***order*** | | ***family*  *(genus)*** | **H** | **L** |  | **H** | **L** |  | **H** | **L** |  | **H** | **L** |  | **H** | **L** |  | **H** | **L** |  |  | **H** | **L** | |  |
| OTU78_16S_ | | *Gammaproteobacteria* | | | *Pasteurellales* | | *Pasteurellaceae* | 0.03 | 0.02 |  | 0.01 | 0.04 |  | - | 0.04 |  | - | 0.03 |  | 0.03 | 0.03 |  | 0.01 | 0.03 |  |  | 0.01 | - |  | |
| OTU79_16S_ | | *Phycisphaerae* | | | n.a. | | n.a. | 0.46 | 0.53 |  | 0.32 | 0.17 |  | 0.18 | 0.11 |  | 0.04 | 0.10 |  | 0.13 | 0.07 |  | 0.04 | 0.16 |  |  | 0.13 | 0.07 |  | |
| OTU80_16S_ | | *Planctomycetes* | | | *Pirellulales* | | *Pirellulaceae* | 0.53 | 0.62 |  | 0.26 | 0.55 |  | 0.59 | 0.27 |  | 0.04 | 0.22 |  | 0.20 | 0.19 |  | 0.18 | 0.22 |  |  | 0.13 | 0.14 |  | |
| OTU81_16S_ | | *Planctomycetes* | | | *Planctomycetales* | | *Planctomycetaceae* | 0.97 | 1.26 |  | 0.44 | 0.82 |  | 0.87 | 0.63 |  | 0.27 | 0.71 |  | 0.52 | 0.55 |  | 0.06 | 0.62 |  |  | 0.36 | 0.81 |  | |
| OTU82_16S_ | | *Firmicutes* | | | *Bacillales* | | *Planococcaceae* | 0.06 | - |  | 0.04 | 0.04 |  | 0.05 | 0.06 |  | - | 0.06 |  | 0.02 | 0.01 |  | 0.05 | - |  |  | 0.08 | 0.03 |  | |
| OTU83_16S_ | | *Actinobacteria* | | | *Actinomycetales* | | *Propionibacteriaceae* | - | - |  | - | - |  | - | - |  | - | - |  | 0.02 | - |  | - | - |  |  | - | - |  | |
| OTU84_16S_ | | *Gammaproteobacteria* | | | *Pseudomonadales* | | *Pseudomonadaceae* | - | - |  | 0.01 | - |  | - | - |  | - | 0.01 |  | - | - |  | - | - |  |  | - | - |  | |
| OTU85_16S_ | | *Actinobacteria* | | | *Actinomycetales* | | *Pseudonocardiaceae* | 3.35 | 3.56 |  | 1.39 | 3.88 |  | 3.65 | 4.45 |  | 3.50 | 4.94 |  | 3.39 | 4.72 |  | 5.30 | 4.35 | **+** |  | 3.10 | 5.09 |  | |
| OTU86_16S_ | | *Alphaproteobacteria* | | | *Rhizobiales* | | *Rhizobiaceae* | 0.03 | 0.02 |  | 0.01 | 0.06 |  | 0.01 | 0.04 |  | 0.04 | 0.03 |  | 0.07 | 0.09 |  | 0.01 | 0.02 |  |  | 0.03 | 0.07 |  | |
| OTU87_16S_ | | *Betaproteobacteria* | | | *Rhodocyclales* | | *Rhodocyclaceae* | 0.03 | 0.03 |  | 0.02 | 0.04 |  | - | 0.06 |  | 0.02 | 0.03 |  | 0.02 | 0.04 |  | 0.05 | 0.02 |  |  | 0.01 | 0.05 |  | |
| OTU88_16S_ | | *Alphaproteobacteria* | | | *Rhodospirillales* | | *Rhodospirillaceae* | 0.03 | 0.02 |  | 0.03 | 0.06 |  | 0.02 | 0.02 |  | 0.05 | 0.02 |  | 0.03 | - |  | 0.01 | 0.01 |  |  | 0.05 | - |  | |
| OTU89_16S_ | | *Alphaproteobacteria* | | | *Ricketsiales* | | *Rickettsiaceae* | 0.01 | 0.01 |  | - | - |  | - | 0.01 |  | - | - |  | - | - |  | - | - |  |  | - | - |  | |
| OTU91_16S_ | | *Actinobacteria* | | | *Micrococcales* | | *Sanguibacteraceae* | 0.02 | 0.02 |  | 0.01 | - |  | 0.02 | - |  | - | 0.01 |  | - | 0.01 |  | 0.02 | 0.01 |  |  | - | 0.02 |  | |
| OTU92_16S_ | | *Bacteroidetes* | | | *Sphingobacteriales* | | *Saprospiraceae* | - | 0.02 |  | - | - |  | - | - |  | - | - |  | - | - |  | - | - |  |  | - | - |  | |
| OTU94_16S_ | | S-BQ2-57 | | | n.a. | | n.a. | 0.02 | - |  | 0.01 | 0.02 |  | - | - |  | - | - |  | - | - |  | - | 0.01 |  |  | - | - |  | |
|  | |  |  | | | | **treatment^a .^** | **^12^CH_3_OH** | |  | **^13^CH_3_OH** | |  | **^12^CH_3_Cl** | |  | **^13^CH_3_Cl** | |  | **^12^CH_3_OH &  ^12^CH_3_Cl** | |  | **^12^CH_3_OH &  ^12^CH_3_Cl** | |  |  | **^12^CH_3_OH &  ^13^CH_3_Cl** | | |  |
|  | | **phylogenetic affiliation^b^** | | | | | | **%-abundance in fraction^c^** | | | | | **^13^C-label^d^** | **%-abundance in fraction^c^** | | | | | **^13^C-label^d^** | **%-abundance in fraction^c^** | | | | | **^13^C-label^d^** |  | **%-ab. fract. ^c^** | | | **^13^C-label^d^** |
| **OTU** | | ***phylum* / *class* or  environmental group** | | ***order*** | | ***family*  *(genus)*** | | **H** | **L** |  | **H** | **L** |  | **H** | **L** |  | **H** | **L** |  | **H** | **L** |  | **H** | **L** |  |  | **H** | **L** | |  |
| OTU95_16S_ | | Sc-NB04 | | n.a. | | n.a. | | 0.04 | 0.11 |  | 0.04 | 0.13 |  | 0.06 | 0.18 |  | - | 0.13 |  | 0.05 | 0.08 |  | - | 0.12 |  |  | 0.04 | 0.17 |  | |
| OTU96_16S_ | | Sediment-1 | | n.a. | | n.a. | | - | - |  | - | - |  | - | - |  | 0.02 | - |  | - | - |  | - | - |  |  | - | - |  | |
| OTU97_16S_ | | *Gammaproteobacteria* | | *Nevskiales* | | *Sinobacteraceae* | | 0.28 | 0.18 |  | 0.07 | 0.17 |  | 0.43 | 0.14 |  | 0.14 | 0.09 |  | 0.15 | 0.11 |  | 0.07 | 0.07 |  |  | 0.09 | 0.10 |  | |
| OTU98_16S_ | | *Acidobacteria* | | *Solibacterales* | | n.a. | | 0.27 | 0.14 |  | 0.14 | 0.08 |  | 0.13 | 0.12 |  | 0.04 | 0.05 |  | 0.15 | 0.08 |  | 0.05 | 0.08 |  |  | 0.08 | 0.07 |  | |
| OTU99_16S_ | | *Actinobacteria* | | *Solirubrobacterales* | | n.a. | | 0.14 | 0.15 |  | 0.12 | 0.06 |  | 0.37 | 0.13 |  | 0.27 | 0.16 |  | 0.27 | 0.15 |  | 0.15 | 0.13 |  |  | 0.23 | 0.15 |  | |
| OTU100_16S_ | | *Bacteroidetes* | | *Sphingobacteriales* | | *Sphingobacteriaceae* | | 0.54 | 0.16 |  | 0.24 | 0.08 |  | 0.31 | 0.10 |  | 0.09 | 0.05 |  | 0.34 | 0.02 |  | 0.11 | 0.09 |  |  | 0.12 | 0.03 |  | |
| OTU101_16S_ | | *Alphaproteobacteria* | | *Sphingomonadales* | | *Sphingomonadaceae* | | 0.01 | - |  | 0.01 | - |  | 0.01 | - |  | 0.02 | 0.01 |  | 0.03 | - |  | 0.01 | 0.01 |  |  | 0.04 | - |  | |
| OTU102_16S_ | | *Firmicutes* | | *Bacillales* | | *Staphylococcaceae* | | 0.08 | 0.02 |  | 0.04 | 0.04 |  | 0.02 | 0.01 |  | - | 0.04 |  | 0.18 | - |  | 0.04 | 0.03 |  |  | 0.01 | 0.03 |  | |
| OTU104_16S_ | | *Actinobacteria* | | *Actinomycetales* | | *Streptomycetaceae* | | 18.27 | 14.48 |  | 11.38 | 20.05 |  | 25.99 | 23.14 |  | 29.94 | 28.31 |  | 26.19 | 26.01 |  | 23.75 | 26.88 |  |  | 26.13 | 28.52 |  | |
| OTU105_16S_ | | Sva0725 | | n.a. | | n.a. | | - | 0.02 |  | - | - |  | 0.01 | - |  | - | 0.01 |  | - | 0.01 |  | - | - |  |  | - | - |  | |
| OTU106_16S_ | | *Chloroflexi* | | *Thermobaculales* | | *Thermobaculaceae* | | - | - |  | - | - |  | - | 0.01 |  | - | - |  | - | - |  | - | - |  |  | - | - |  | |
| OTU107_16S_ | | TM6 | | n.a. | | n.a. | | 0.06 | 0.01 |  | 0.04 | - |  | - | 0.01 |  | - | 0.02 |  | - | - |  | - | 0.02 |  |  | 0.00 | - |  | |
| OTU108_16S_ | | *"Candidatus* Saccharibacteria*"*    TM7 | | n.a. | | n.a. | | 1.16 | 0.84 |  | 0.34 | 0.40 |  | 1.67 | 1.51 |  | 0.77 | 1.22 |  | 0.99 | 0.34 |  | 0.33 | 0.92 |  |  | 1.32 | 0.56 | **+** | |
| OTU110_16S_ | | *Firmicutes* | | *Veillonellales* | | *Veillonellaceae* | | - | 0.01 |  | - | - |  | - | - |  | - | - |  | - | 0.01 |  | - | - |  |  | 0.00 | - |  | |

|  |  |  | | **treatment^a .^** | **^12^CH_3_OH** | |  | **^13^CH_3_OH** | |  | **^12^CH_3_Cl** | |  | **^13^CH_3_Cl** | |  | **^12^CH_3_OH &  ^12^CH_3_Cl** | |  | **^12^CH_3_OH &  ^12^CH_3_Cl** | |  |  | **^12^CH_3_OH &  ^13^CH_3_Cl** | | |  |
| --- | --- | --- | --- | --- | --- | --- | --- | --- | --- | --- | --- | --- | --- | --- | --- | --- | --- | --- | --- | --- | --- | --- | --- | --- | --- | --- | --- |
|  | **phylogenetic affiliation^b^** | | | | **%-abundance in fraction^c^** | | | | | **^13^C-label^d^** | **%-abundance in fraction^c^** | | | | | **^13^C-label^d^** | **%-abundance in fraction^c^** | | | | | **^13^C-label^d^** |  | **%-ab. fract. ^c^** | | | **^13^C-label^d^** |
| **OTU** | ***phylum* / *class* or  environmental group** | | ***order*** | ***family*  *(genus)*** | **H** | **L** |  | **H** | **L** |  | **H** | **L** |  | **H** | **L** |  | **H** | **L** |  | **H** | **L** |  |  | **H** | **L** | |  |
| OTU111_16S_ | *Verrucomicrobia* | | *Verrucomicrobiales* | *Verrucomicrobiaceae* | 0.01 | 0.02 |  | 0.01 | - |  | 0.01 | - |  | 0.02 | - |  | 0.02 | - |  | - | - |  |  | 0.00 | - |  | |
| OTU112_16S_ | W4 | | n.a. | n.a. | 0.67 | 1.13 |  | 0.32 | 0.96 |  | 0.43 | 0.57 |  | 0.21 | 0.64 |  | 0.62 | 0.65 |  | 0.11 | 0.62 |  |  | 0.21 | 0.52 |  | |
| OTU113_16S_ | *Bacteroidetes* | | *Flavobacteriales* | *Weeksellaceae* | - | - |  | - | - |  | - | - |  | - | - |  | - | - |  | - | - |  |  | 0.01 | - |  | |
| OTU114_16S_ | WMSP1 | | n.a. | n.a. | - | - |  | - | - |  | - | - |  | - | 0.02 |  | 0.02 | - |  | - | - |  |  | 0.01 | 0.03 |  | |
| OTU115_16S_ | WPS-2 | | n.a. | n.a. | 0.09 | 0.05 |  | 0.05 | 0.02 |  | 0.14 | 0.05 |  | 0.05 | 0.04 |  | 0.12 | 0.04 |  | 0.10 | 0.08 |  |  | 0.07 | 0.07 |  | |
| OTU116_16S_ | *Gammaproteobacteria* | | *Xanthomonadales* | *Xanthomonadaceae* | 10.99 | 10.04 |  | 2.09 | 4.66 |  | 6.52 | 6.19 |  | 4.37 | 6.70 |  | 5.37 | 7.05 |  | 4.28 | 6.92 |  |  | 6.64 | 8.66 |  | |
|  |  | |  |  |  |  |  |  |  |  |  |  |  |  |  |  |  |  |  |  |  |  |  |  |  |  | |

| \|  \|  \|  \| \| **treatment^a .^** \| **^12^CH_3_OH** \| \|  \| **^13^CH_3_OH** \| \|  \| **^12^CH_3_Cl** \| \|  \| **^13^CH_3_Cl** \| \|  \| **^12^CH_3_OH &  ^12^CH_3_Cl** \| \|  \| **^12^CH_3_OH &  ^12^CH_3_Cl** \| \|  \|  \| **^12^CH_3_OH &  ^13^CH_3_Cl** \| \|  \| \| --- \| --- \| --- \| --- \| --- \| --- \| --- \| --- \| --- \| --- \| --- \| --- \| --- \| --- \| --- \| --- \| --- \| --- \| --- \| --- \| --- \| --- \| --- \| --- \| --- \| --- \| --- \| \|  \| **phylogenetic affiliation^b^** \| \| \| \| **%-abundance in fraction^c^** \| \| \| \| \| **^13^C-label^d^** \| **%-abundance in fraction^c^** \| \| \| \| \| **^13^C-label^d^** \| **%-abundance in fraction^c^** \| \| \| \| \| **^13^C-label^d^** \|  \| **%-ab. fract. ^c^** \| \| **^13^C-label^d^** \| \| **OTU** \| ***phylum* / *class* or  environmental group** \| \| ***order*** \| ***family*  *(genus)*** \| **H** \| **L** \|  \| **H** \| **L** \| **H** \| **L** \|  \| **H** \| **L** \| **H** \| **L** \|  \| **H** \| **L** \|  \| **H** \| **L** \|   ***cmuA* phylotypes** | | | | | | | | | | | | | | | | | | | | | | | | | |
| --- | --- | --- | --- | --- | --- | --- | --- | --- | --- | --- | --- | --- | --- | --- | --- | --- | --- | --- | --- | --- | --- | --- | --- | --- | --- | --- | --- | --- | --- | --- | --- | --- | --- | --- | --- | --- | --- | --- | --- | --- | --- | --- | --- | --- | --- | --- | --- | --- | --- | --- | --- | --- | --- | --- | --- | --- | --- | --- | --- | --- | --- | --- | --- | --- | --- | --- | --- | --- | --- | --- | --- | --- | --- | --- | --- | --- | --- | --- | --- | --- | --- | --- | --- | --- | --- | --- | --- | --- | --- | --- | --- | --- | --- | --- | --- | --- | --- | --- | --- | --- | --- | --- |
| OTU1_cmuA_ | *Alphaproteobacteria* | n.a. | n.a. | - | - |  | - | - |  | 7.03 | 6.69 |  | 6.12 | 1.09 |  | - | 0.12 |  | - | 0.08 |  |  | 0.48 | - |  |
| OTU2_cmuA_ | *Alphaproteobacteria* | *Rhizobiales* | *Methylobacteriaceae  (Methylobacterium)* | 34.96 | 70.33 |  | 64.05 | 71.40 | **+** | 11.89 | 56.82 |  | 63.76 | 63.64 | **+** | 51.84 | 80.07 |  | 42.67 | 78.55 |  |  | 83.75 | 56.53 | **+** |
| OTU3_cmuA_ | *Alphaproteobacteria* | *Rhizobiales* | *Methylobacteriaceae  (Methylobacterium)* | 3.25 | 23.82 |  | 26.62 | 14.42 | **+** | 15.68 | 27.86 |  | 23.96 | 18.91 |  | 13.88 | 16.37 |  | 18.24 | 18.40 | **+** |  | 12.92 | 30.31 |  |
| OTU4_cmuA_ | *Alphaproteobacteria* | *Rhizobiales* | *Methylobacteriaceae  (Methylobacterium)* | 2.44 | 5.28 |  | 7.42 | 3.20 | **+** | 4.86 | 7.53 |  | 3.97 | 9.27 |  | 1.95 | 3.02 |  | 3.26 | 2.93 | **+** |  | 1.43 | 11.12 |  |
| OTU5_cmuA_ | *Alphaproteobacteria* | *Rhizobiales* | *Methylobacteriaceae  (Methylobacterium)* | - | 0.49 |  | - | 8.47 |  | 0.54 | 0.91 |  | 0.14 | 5.82 |  | 0.43 | 0.36 |  | - | 0.04 |  |  | - | 1.95 |  |
| OTU6_cmuA_ | *Alphaproteobacteria* | *Rhizobiales* | *Hyphomicrobiaceae  (Hyphomicrobium)* | 59.35 | - |  | 1.91 | 1.37 |  | 60.00 | 0.06 |  | 2.06 | 1.09 |  | 31.67 | 0.06 |  | 35.18 | - | **+** |  | 1.43 | 0.09 |  |
| OTU7_cmuA_ | *Firmicutes* | n.a. | n.a. | - | 0.08 |  | - | 1.14 |  | - | 0.13 |  | - | 0.18 |  | - | - |  | - | - |  |  | - | - |  |
| OTU8_cmuA_ | *Alphaproteobacteria* | *Rhizobiales* | *Methylobacteriaceae  (Methylobacterium)* | - | - |  | - | - |  | - | - |  | - | - |  | 0.22 | - |  | 0.65 | - | **+** |  | - | - |  |
|  |  |  |  |  |  |  |  |  |  |  |  |  |  |  |  |  |  |  |  |  |  |  |  |  |  |
| ***mdh* phylotypes  (*mxaF*/*xoxF*)** | | | | | | | | | | | | | | | | | | | | | | | | | |
| OTU1_mdh_ | *Alphaproteobacteria* | *Rhizobiales* | *Bradyrhizobiaceae  (Bradyrhizobium)* | 3.20 | 2.90 |  | 2.56 | 2.26 |  | 1.86 | 2.70 |  | 0.59 | 2.04 |  | 0.35 | 2.27 |  | 2.21 | 2.26 | **+** |  | 2.28 | 2.47 | **+** |
| OTU2_mdh_ | *Alphaproteobacteria* | *Rhizobiales* | *Hyphomicrobiaceae  (Hyphomicrobium)* | 0.29 | 1.18 |  | 4.60 | 1.16 | **+** | 0.55 | 1.36 |  | 2.56 | 1.36 | **+** | 3.01 | 1.69 |  | 1.59 | 2.07 |  |  | 2.03 | 2.31 |  |
| OTU3_mdh_ | *Alphaproteobacteria* | *Rhizobiales* | *Methylobacteriaceae  (Methylobacterium)* | 0.57 | 0.08 |  | 12.68 | 0.03 | **+** | 0.13 | 0.02 |  | - | 0.13 |  | - | 0.09 |  | 0.29 | 0.04 |  |  | 0.21 | 0.05 |  |

|  |  |  | | **treatment^a .^** | **^12^CH_3_OH** | |  | **^13^CH_3_OH** | |  | **^12^CH_3_Cl** | |  | **^13^CH_3_Cl** | |  | **^12^CH_3_OH &  ^12^CH_3_Cl** | |  | **^12^CH_3_OH &  ^12^CH_3_Cl** | |  |  | **^12^CH_3_OH &  ^13^CH_3_Cl** | | |  |
| --- | --- | --- | --- | --- | --- | --- | --- | --- | --- | --- | --- | --- | --- | --- | --- | --- | --- | --- | --- | --- | --- | --- | --- | --- | --- | --- | --- |
|  | **phylogenetic affiliation^b^** | | | | **%-abundance in fraction^c^** | | | | | **^13^C-label^d^** | **%-abundance in fraction^c^** | | | | | **^13^C-label^d^** | **%-abundance in fraction^c^** | | | | | **^13^C-label^d^** |  | **%-ab. fract. ^c^** | | | **^13^C-label^d^** |
| **OTU** | ***phylum* / *class* or  environmental group** | | ***order*** | ***family*  *(genus)*** | **H** | **L** |  | **H** | **L** |  | **H** | **L** |  | **H** | **L** |  | **H** | **L** |  | **H** | **L** |  |  | **H** | **L** | |  |
| OTU4_mdh_ | *Alphaproteobacteria* | | *Rhizobiales* | *Beijerinckiaceae  (Methyloligni/-ferula )* | - | 0.04 |  | 0.26 | 0.09 |  | 0.03 | - |  | 0.08 | 0.10 |  | - | - |  | - | 0.06 |  |  | 0.02 | 0.09 |  | |
| OTU5_mdh_ | *Alphaproteobacteria* | | *Rhizobiales* | *Beijerinckiaceae  (Methylocella)* | 0.36 | 0.30 |  | 0.17 | 0.31 |  | 0.33 | 0.24 |  | 0.32 | 0.45 |  | 0.54 | 0.32 |  | 0.20 | 0.56 |  |  | 0.41 | 0.40 |  | |
| OTU6_mdh_ | *Alphaproteobacteria* | | *Rhizobiales* | *Beijerinckiaceae  (Methylocella)* | 0.35 | 0.11 |  | 0.13 | 0.55 |  | 0.32 | 0.24 |  | 0.16 | 0.34 |  | 0.48 | 0.17 |  | 0.08 | 0.45 |  |  | 0.38 | 0.49 |  | |
| OTU7_mdh_ | *Betaproteobacteria* | | n.a. | n.a. | 0.01 | - |  | - | 0.18 |  | 0.22 | 0.04 |  | 0.24 | 0.08 |  | 0.16 | 0.06 |  | 0.12 | 0.09 |  |  | 0.02 | 0.14 |  | |
| OTU8_mdh_ | *Alphaproteobacteria* | | *Rhizobiales* | *Beijerinckiaceae  (Methyloligni/-ferula )* | 0.01 | 0.15 |  | - | 0.12 |  | 0.02 | 0.06 |  | - | 0.18 |  | 0.08 | 0.06 |  | 0.08 | 0.15 |  |  | - | 0.21 |  | |
| OTU9_mdh_ | *Alphaproteobacteria* | | *Rhizobiales* | *Hyphomicrobiaceae  (Hyphomicrobium)* | 0.19 | 0.11 |  | 0.13 | 0.09 |  | 0.07 | 0.06 |  | 0.39 | 0.16 |  | 0.30 | 0.15 |  | - | 0.11 |  |  | 0.06 | 0.14 |  | |
| OTU10_mdh_ | *Alphaproteobacteria* | | *Rhizobiales* | *Hyphomicrobiaceae  (Hyphomicrobium)* | 0.04 | 0.04 |  | 0.04 | 0.27 |  | 0.03 | - |  | 0.12 | 0.05 |  | 0.05 | 0.11 |  | 0.16 | 0.09 |  |  | 0.06 | 0.09 |  | |
| OTU11_mdh_ | *Alphaproteobacteria* | | *Rhizobiales* | *Beijerinckiaceae  (Methylocella)* | - | 0.04 |  | 0.78 | - | **+** | 0.06 | 0.02 |  | 0.04 | - |  | 0.03 | 0.02 |  | 0.37 | 0.02 |  |  | 0.04 | 0.02 |  | |
| OTU12_mdh_ | *Alphaproteobacteria* | | *Rhizobiales* | *Beijerinckiaceae  (Methylocella)* | 0.09 | 0.15 |  | 0.22 | 0.03 |  | 0.14 | 0.08 |  | - | 0.21 |  | - | 0.15 |  | 0.20 | 0.13 |  |  | 0.19 | 0.14 |  | |
| OTU13_mdh_ | *Betaproteobacteria* | | n.a. | n.a. | 0.04 | - |  | - | - |  | 0.01 | - |  | - | - |  | - | - |  | - | - |  |  | - | - |  | |
| OTU14_mdh_ | *Alphaproteobacteria* | | *Rhodospirillales* | *Acetobacteriaceae  (Acidiphilium)* | 0.32 | 0.38 |  | 11.47 | 0.09 | **+** | 0.13 | 0.04 |  | 0.12 | 0.24 |  | - | 0.15 |  | 0.37 | 0.22 |  |  | 0.21 | 0.12 |  | |
| OTU15_mdh_ | *Alphaproteobacteria* | | *Rhizobiales* | *Beijerinckiaceae  (Methyloligni/-ferula )* | 0.01 | - |  | 1.78 | 0.03 | **+** | - | - |  | 0.04 | 0.08 |  | 0.08 | 0.04 |  | - | 0.02 |  |  | 0.09 | 0.07 |  | |
| OTU16_mdh_ | *Alphaproteobacteria* | | *Rhizobiales* | *Beijerinckiaceae  (Methyloferula)* | - | - |  | 0.17 | - |  | - | - |  | - | - |  | - | - |  | 0.04 | - |  |  | - | - |  | |

|  |  |  | | **treatment^a .^** | **^12^CH_3_OH** | |  | **^13^CH_3_OH** | |  | **^12^CH_3_Cl** | |  | **^13^CH_3_Cl** | |  | **^12^CH_3_OH &  ^12^CH_3_Cl** | |  | **^12^CH_3_OH &  ^12^CH_3_Cl** | |  |  | **^12^CH_3_OH &  ^13^CH_3_Cl** | | |  |
| --- | --- | --- | --- | --- | --- | --- | --- | --- | --- | --- | --- | --- | --- | --- | --- | --- | --- | --- | --- | --- | --- | --- | --- | --- | --- | --- | --- |
|  | **phylogenetic affiliation^b^** | | | | **%-abundance in fraction^c^** | | | | | **^13^C-label^d^** | **%-abundance in fraction^c^** | | | | | **^13^C-label^d^** | **%-abundance in fraction^c^** | | | | | **^13^C-label^d^** |  | **%-ab. fract. ^c^** | | | **^13^C-label^d^** |
| **OTU** | ***phylum* / *class* or  environmental group** | | ***order*** | ***family*  *(genus)*** | **H** | **L** |  | **H** | **L** |  | **H** | **L** |  | **H** | **L** |  | **H** | **L** |  | **H** | **L** |  |  | **H** | **L** | |  |
| OTU17_mdh_ | *Alphaproteobacteria* | | *Rhizobiales* | *Rhizobiaceae  (Sinorhizobium)* | 10.08 | 12.04 |  | 6.99 | 11.48 |  | 14.56 | 12.17 |  | 17.90 | 11.32 | **+** | 18.66 | 10.68 |  | 14.22 | 11.64 | **+** |  | 11.82 | 10.23 |  | |
| OTU18_mdh_ | *Alphaproteobacteria* | | *Burkholderiales* | *Burkholderiaceae  (Burkholderia)* | 0.39 | 0.46 |  | 0.39 | 0.49 |  | 0.29 | 0.52 |  | 1.46 | 0.52 | **+** | 1.08 | 0.65 |  | 0.53 | 0.43 | **+** |  | 0.49 | 0.56 |  | |
| OTU19_mdh_ | *Alphaproteobacteria* | | *Rhizobiales* | *Bradyrhizobiaceae  (Bradyrhizobium)* | 0.80 | 1.52 |  | 0.91 | 1.34 |  | 1.98 | 1.16 |  | 1.93 | 1.55 |  | 3.20 | 1.23 |  | 1.39 | 1.08 | **+** |  | 0.87 | 1.03 |  | |
| OTU20_mdh_ | *Alphaproteobacteria* | | *Rhizobiales* | *Hyphomicrobiaceae  (Hyphomicrobium)* | 0.71 | 0.95 |  | 0.22 | 0.85 |  | 0.80 | 0.98 |  | 1.14 | 0.97 | **+** | 1.72 | 1.08 |  | 0.49 | 0.93 |  |  | 0.30 | 1.14 |  | |
| OTU21_mdh_ | *Alphaproteobacteria* | | *Rhizobiales* | *Hyphomicrobiaceae  (Hyphomicrobium)* | 1.98 | 2.78 |  | 8.47 | 4.09 | **+** | 2.45 | 3.56 |  | 6.23 | 4.19 | **+** | 7.26 | 5.15 |  | 4.29 | 4.78 | **+** |  | 5.48 | 6.04 |  | |
| OTU22_mdh_ | *Alphaproteobacteria* | | *Rhizobiales* | *Hyphomicrobiaceae  (Hyphomicrobium)* | 1.18 | 2.55 |  | 0.61 | 3.02 |  | 3.36 | 2.46 |  | 3.12 | 3.09 |  | 4.22 | 2.56 |  | 0.45 | 2.72 |  |  | 1.77 | 2.45 |  | |
| OTU23_mdh_ | *Alphaproteobacteria* | | *Burkholderiales* | *Burkholderiaceae  (Burkholderia)* | 1.01 | 1.07 |  | 1.61 | 1.56 | **+** | 1.35 | 1.36 |  | 2.01 | 1.60 | **+** | 2.90 | 1.19 |  | 0.61 | 1.27 |  |  | 2.11 | 1.19 |  | |
| OTU24_mdh_ | *Alphaproteobacteria* | | *Rhizobiales* | *Bradyrhizobiaceae  (Bradyrhizobium)* | 53.52 | 42.72 |  | 30.76 | 39.44 |  | 35.02 | 41.89 |  | 14.24 | 41.42 |  | 3.39 | 41.89 |  | 32.00 | 41.57 |  |  | 42.93 | 43.72 | **+** | |
| OTU25_mdh_ | *Alphaproteobacteria* | | *Rhizobiales* | *Bradyrhizobiaceae  (Bradyrhizobium)* | 24.82 | 30.41 |  | 15.03 | 32.51 |  | 36.27 | 31.06 |  | 47.32 | 29.92 | **+** | 52.49 | 30.26 |  | 40.29 | 29.31 | **+** |  | 28.21 | 26.92 |  | |

^a^ Abbreviation: CH_3_OH, methanol amended; CH_3_Cl, chloromethane amended; 12, ^12^C isotopologue; 13, ^13^C isotopologue.

^b^ Phylogenetic affiliation is based on SILVA classification for 16S rRNA phylotypes and phylogenetic trees for *cmuA* and *mxaF*/*xoxF* phylotypes.

^c^ Relative abundance based on singleton-clean dataset. -, not detected in the fraction.

^d^ Detected label by ^13^C isotopes is indicated by +.

^e^ This OTU affiliated with plant mitochondrial 16s rRNA genes.

.

**Figure S1.** **CO_2_ formation during the SIP experiment.** CH_3_OH treatment (a), CH_3_Cl treatment (b), treatments with both substrates (c). Red arrows, substrate amendment.

**Figure S2. CsCl buoyant density of gradient fractions (a) and relative distribution of DNA (b)**. Panel a: line, linear regression (y= - 0.0052 + 1.752; R2 = 0.992); Error bars, standard deviations (n = 10). Panel b: light grey backgrounds, assigned light fractions (BD <1.715 g ml^-1^); dark grey background, assigned heavy fractions (BD >1.730 g ml^-1^).

**Figure S3. NMDS analyses of the 16S rRNA gene (a), *cmuA* (b) and *mxaF/xoxF* phylotypes (c) from the SIP experiment.** Relative abundances of 16S rRNA gene sequences based on family-level (defined as 90% sequence similarity), and of *cmuA* and *mxaF/xoxF* sequences based on a similarity cut-off value of 80% and 77%, respectively. Stress values: 0.03 (16S rRNA gene sequences), 0.0 (*cmuA),* and 0.04 (*mxaF/xoxF)* sequences. Blue, control treatments with [^12^C]-isotopologues; red, treatments with [^13^C]-isotopologues; black, combined [^12^C] and [^13^C] replicates. In the case of the combined substrate microcosms, the [^13^C] substrate is noted. Lines, convex polygons including all data for the CH_3_OH or CH_3_Cl treatments, respectively. Dashed lines, convex polygon including all data for the combined substrate treatments (CH_3_OH plus CH_3_Cl).

**Figure S4. Phylogenetic tree of all labelled bacterial phylotypes (16S rRNA gene).** Closed circle, phylogenetic affiliation of all labelled phylotypes. The tree is based on 69 nucleotide sequences in total. The tree focuses on *Actinobacteria* (*Microbacteriaceae*, *Pseudonocardiaceae*), *Beijerinckiaceae* and the “*Candidatus* Saccharibacteria” (also known as “TM7”). Outgroup, 16S rRNA gene of S. *acidocaldarius*. Phyla without labelled phylotypes are collapsed. Bootstrap values refer to 1000 replicates. Dots at the nodes indicate congruent nodes with trees based on the maximum likelihood and maximum parsimony method (●, true for both phylogenetic trees; ●, only true for one phylogenetic tree). The tree includes sequences from the next hit of the BLAST analysis of each phylotype (dashed underlined), known CH_3_Cl-utilizing strains (indicated with ⯁), and a phylotype (OTU438_16S_, LT6079905) identified as an important methylotroph in the previous substrate SIP experiment. If known the isolation origin of a sequence is given in brackets. Accession numbers are given in squared brackets. The bar indicates 0.05 change per nucleotide.

**Figure S5. Part 1.**

**Figure S5. Part 2.**

**Figure S5. Part 3.**

**Figure S5. Phylogenetic tree all *mxaF* and *xoxF* phylotypes obtained.** OTU1_mdh_ to OTU25_mdh_; indicated with ⯁) in a neighbour joining tree. Labelled OTUs are bold (third part of the tree). Further phylotypes has been published in a previous study (Morawe *et al.*, 2017). The tree is based on 352 nucleotide sequences. Sequences from other PQQ-dependent dehydrogenases served as outgroup. Bootstrap values were calculated from 100 replicates, values ≥50 are presented. Dots at the nodes indicate congruent nodes with trees based on the maximum likelihood and maximum parsimony method (●, congruent in all phylogenetic trees; ●, only congruent with one phylogenetic tree). The bar indicates 0.05 difference per nucleotide. The tree includes known *mxaF* and *xoxF* sequences of several bacteria and from genomic annotations. Phylogenetic affiliation is indicated by the same font colour and coloured boxes (i.e., *Methylobacteriaceae*; *Beijerinckiaceae; Methylocystaceae; Hyphomicrobiaceae; Burkholderiaceae; Bradyrhizobiaceae;* unclassified *Betaproteobacteria*). The tree includes also sequences of uncultured bacteria from different studies focussing on methylotrophs. The origin of the sequences as well as the study is given in brackets (i.e., M_2002_, (Morris *et al.*, 2002); M_2005_, (Moosvi *et al.*, 2005); R_2000_, (Radajewski *et al.*, 2000); R_2002_, (Radajewski *et al.*, 2002); S, (Stacheter *et al.*, 2013); T, (Taubert *et al.*, 2015)).

Accession numbers of sequences are given in squared brackets. In the case of condensed branches, the number of sequences (in bold) and the accession numbers are either directly given in squared brackets or listed below when condensed branch name are given (see below):

*mxaF*

- uncultured bacteria (oak forest soil, R_2002_) [6: AY080930; AY080932; AY080933; AY080934; AY080935; AY080936]
- *Methylocystaceae* - *Methylopila* [5: AJ878071; JQ582798; KP407883; JX134090; JX134089]
- *Methylocystaceae* - *Hansschlegelia* [2: DQ652143; DQ652144]
- *Methylococcaceae* [12: *Methylohalobius* [NZ_ATXB01000001]; *Methylocaldum* [KE386490]; *Methylococcus* [NC_002977]; *Methylomicrobium* [3: NC_016112; NZ_KB455575; CM001475]; *Methylovulum* [NZ_KB913025]; *Methylobacter* [JX312967]; *Methylomonas* [NC_015572]; *Methylosarcina* [NZ_KB889965]; *Methylobacter* [2:NZ_JH109154; KB912877]]

*xoxF* 4 *(Methylophilaceae)*

(a) CP001672; NC_014207; NZ_KB905141; NZ_KB905146; NZ_AAUX01000001; DS995299; NC_007947; NC_012969; CP001672; NC_014207; NC_012969; NZ_KB905145; NC_007947; NC_012969; NC_014207; NC_007947

*xoxF* 2 (*Verrucomicrobia)*

1. CP002221; NC_010794; NZ_CAHT01000021; NC_013260; DQ084247

*xoxF* 3 (*Rhizobiales,* some *Betaproteobacteria,* and *Gammaproteobacteria)*

(c ) NC_018485; NZ_KB889963; NZ_CAFK01000299; NC_008536; NZ_KB912877; KE386490; NC_007947; NC_011666; NZ_ARWA01000001; NC_012791; CP000741; NC_011894; CP002279; NC_016617

*xoxF* 1 (*Xanthomonas* and *Beijerinckiaceae*)

(d) NC_003919; NZ_AEQX01000324; NC_010688; NC_013260; NC_011666; NZ_ARWA01000001

*xoxF* 5 (various *Alphaproteobacteria, Betaproteobacteria* and *Gammaproteobacteria*)

(e) *Burkholderia* sp. [6: NC_015137; NC_007952; NC_016625; NC_010625; CP012748; CP002014]

(f) *Methylophaga* [5: NZ_GG657899; NC_017857; CP003380; NZ_APHR01000021; NZ_APHR01000099]

(g) *Methylococcaceae* [8: NC_016112; NZ_KB455575; CM001475; NZ_KB889965; NZ_KB912877; NC_015572; NZ_KB913025]

(h) *Rhodobacteraceae* [8: CH902584; ABCL01000006; NZ_DS022277; NC_008686; NC_022041; NZ_AAYA01000026; NC_008209; NC_015730]

(k) *Methylobacterium* [8: CP000943; AP014809; CP001298; NC_012808; NC_011894; CP017640; CP001001; ANPA01000003]

(l) *Methyloversatilis* [4: NZ_AFHG01000059; NZ_ARVV01000001; NZ_KB900539; EU548065]

Outgroup (PQQ-dependent dehydrogenases)

NC_008825; AF355798; CP000555; AF326086; JN808865; EU548063; EU548066

**Figure S6. Relative abundances of bacterial 16S rRNA phylotypes.** Boxes, Families considered as being ^13^C-labelled.

**Supplemental Information on Materials & Methods**

#### Design of new primers targeting *cmuA* in the environment

PCR amplification targeting *cmuA* in samples from environments such as soil, marine ecosystems, and phyllosphere were performed previously using primers targeting the end of the methyltransferase domain of the CmuA protein of chloromethane dehalogenase (Miller *et al.*, 2004; Borodina *et al.*, 2005; Nadalig *et al.*, 2011). These studies and sequencing of new bacterial genomes with *cmuA* genes enriched the database allowing the design of new primers that target the region between the two conserved domains (IPR006360 and IPR006158 for methyltransferase and corrinoid binding domains, respectively) of protein CmuA. A total of 113 *cmuA* sequences were retrieved from the NCBI database after a BLAST search using the *M. extorquens* *cmuA* (Mchl_5697) sequence. Of those, the most divergent CmuA-like protein of *Vibrio orientalis* ATCC33934 (VIA_000821, Uniprot) was excluded as the strain was unable to dechlorinate chloromethane (L. Besaury and F. Bringel, unpublished data). Sequences were aligned using clustalW-multialign in Mobyle (http://mobyle.pasteur.fr/cgi-bin/portal.py#forms::clustalw-multialign alignment). Based on the alignment of 112 *cmuA* sequences, new sets of primers were designed. Nonetheless, these primers come with a caveat that is the remaining possible limitation in the amplification of a few known divergent sequences. Basing on sequence divergence (Table S2), primer cmuAr422 would poorly enable the PCR amplification of *cmuA*-related genes found in genomes of two exclusively anaerobic bacteria of *Desulfomonile tiedjei* (Deltaproteobacteria) and *Thermosediminibacter oceani* (Clostridia) (Nadalig *et al.* 2014), which may not be relevant for this study performed in aerobiosis. The new and old primers were tested in comparative PCR amplification assays (Figure S7).





Figure S7. Comparing two primers sets targeting *cmuA.* PCR amplification of *cmuA* targets the region between conserved domains (in black) IPR006360 and IPR006158 of the encoded protein CmuA. (A) Position of primer box 1 (Miller *et al.*, 2004) and primer box 2 (This study, Table S3). Name, localization and orientation (arrow) of each primer are shown. Numbers indicate PCR product size in bp. (B) PCR-amplified *cmuA* products using reference genomic DNA and environmental DNA. Amplifications were performed using 10 ng DNA. Tracks 1-3, DNA from chloromethane-degrading strains, including the negative control *Leisingera methylohalidovorans* (track 1) (lacking *cmuA*, Schäfer *et al*., 2004); *M. extorquens* CM4 (2) and *Hyphomicrobium* sp. strain MC1 (3) are positive controls. Tracks 4-6, environmental DNA from a native forest soil (4), a [^12^C]-CH_3_Cl pre-incubated forest soil (5), and a forest soil incubated with [^13^C]-CH_3_Cl (6). (C) Sensitivity of two sets of primers sets, in PCR amplification of *cmuA* using DNA from isolated chloromethane-degrading strains including the negative control *L. methylohalidovorans* and a forest soil incubated with ^13^CH_3_Cl (6). Amplifications were more specific with the new primers cmuAf422/cmuAr422 (Figure S5 box 2) than with cmuA802F/cmuA1244R (box 1). PCR with primer set cmuA802F/cmuA1244 (Figure S5 box 1) was performed as previously (Miller *et al.*, 2004). The PCR program for primer set cmuAf422/cmuAr422 (Figure S5, box 2) involves denaturation for 10 min at 95°C, followed by 30 cycles of denaturation for 15 seconds at 94°C followed by annealing/elongation for 60 seconds at 56°C, and post-elongation for 5 minutes at 72°C. The new primers were validated on a forest soil under 3 different conditions (native, [^12^C]-CH_3_Cl and [^13^C]-CH_3_Cl pre-incubated, and results were compared with those obtained with the original primers (Figure S5).

**Design of new primers targeting environmental methanol dehydrogenases.** New primers that target both *mxaF* and the phylogenetically related *xoxF* gene were designed. Members of 3 of known XoxF clades (i.e. XoxF3, XoxF4 and XoxF5, Keltjens et al., 2014) which represent, to the best of our knowledge, all clades so far identified in sequenced genomes of CH_3_Cl-degraders (Nadalig *et al*. 2014), were considered in primer design. Not considering members of the less common XoxF1 and XoxF2 may represent a potential limitation for detecting the detection of these 2 clades using the new set of mdh primers. For primer design, a total of 120 *mxaF/xoxF* sequences, mostly found in sequenced genomes, were aligned using clustalW-multialign (Larkin *et al.* 2007) within Mobyle (http://mobyle.pasteur.fr/cgi-bin/portal.py#forms::clustalw-multialign alignment). Aligned sequences include complete sets of multiple *xoxF* copies commonly encountered in genomes of methylotrophic bacteria, including from dechlorinating strains (*Methylobacterium* *extorquens* strain CM4 and *Hyphomicrobium* sp. strain MC1) (**List 2**). Based on the obtained alignment, primer 1555R previously used to target *mxaF* in soil (Stacheter *et al.*, 2013) would poorly detect *xoxF* (Figure S8), whereas the newly designed primer set targets all considered *mxaF* and *xoxF.*

**Figure S8. Rationale for design of new primers targeting methanol dehydrogenases (mdh) encoded by *mxaF*/*xoxF*.** Consensus sequences were generated using WebLogo (Crooks *et al*. 2004). An equimolar mixture of the two mdh1 and mdh2 forward primers, which differ by a single C/G substitution, were designed to compensate for the less conserved internal targeted hybridization loci.

The newly designed forward mdh primers are shorter compared to the previous primer 1003f, which displays a weakly conserved 3’-end, a crucial position for successful DNA elongation of a hybridized primer. As an example, the 3’-end T of primer 1003f would strongly disfavor PCR amplification of divergently-ending sequences such as 4 out of the 5 *xoxF* homologs of the chloromethane-degrader *Hyphomicrobium* sp. MC1 (i.e. HYPMCv2_3613 *xoxF3*; HYPMCv2_0800 *xoxF5*; HYPMCv2_4120 *xoxF5*; HYPMCv2_1896 *xoxF*), the 3 *xoxF5* genes of the dichloromethane-degrading *Hyphomicrobium denitrificans* ATCC 51888 (Hden_2848; Hden_1305; Hden_1617), as well as genes of non-dechlorinating methylotrophs such as *Hyphomicrobium nitrativorans* NL23 (W911_13290 *xoxF*), *Methylocella silvestris* BL2 (Msil_2260; Msil_3387 *xoxF3*), *Methylobacterium extorquens* PA1 (Mext_0099 *xoxF3*), *Methylobacterium nodulans* strain ORS2060 (Mnod_2344 *xoxF3*) and *Methylobacillus flagellatus* KT (Mfla_1451 *xoxF3*).

**PCR amplification of 16S rRNA and *mxaF*/*xoxF* genes**. PCR reactions were carried out in 0.2 mL microcentrifuge tubes in a LabCycler thermal cycler (SensoQuest, Göttingen, Germany). Obtained PCR products were checked by agarose gel electrophoresis.

For 16S rRNA amplification, reaction mix (50 µL) consisted of 23.75 µL distilled water, 5 µL DNA template solution (between 1.5 - 15 ng), 2.5 µL of 10 µM degenerate primer stocks 341for and 785/805rev (Table 2) using Crystal Taq DNA polymerase (Biolab products GmbH, Lüneburg, Germany) as recommended by the supplier. After initial denaturation (95°C, 5 min), DNA amplification was achieved by 40 cycles of 1 min steps of denaturation at 95°C, annealing at 55°C, and extension at 72°C, followed by a final extension step of 5 min at 72°C.

Amplification of the *cmuA* gene was performed as follows. A reaction mix of 5 µL of 10x buffer provided by the supplier (1x: Tris- HCl 10 mM, KCl 50 mM, MgCl2 1.5 mM, Triton 0.1 %, BSA 0.2 mg. mL-1, pH 9.0, MP Biomedicals) 0.5 µL dNTPs (20 mM), 3.25 µL forward primer cmuAf422 and 0.75 µL of the reverse primer cmuAr422 (Table 4.2) as 20 µM stocks, 5 µL DNA template solution (between 1.5 - 15 ng), and 33.5 µL of distilled water was prepared. After initial denaturation (95°C, 10 min), 2 µL TAQ polymerase mix (prepared as 2 µL of TAQ polymerase (5U/µL, MP biomedicals) added to 1 µL of 10x buffer and 7 µL H_2_O) was added to the reaction mix. DNA amplification was performed for 30 cycles consisting of 15 s denaturation at 95°C, annealing/extension for 30 s at 56°C, followed by a final extension step of 5 min at 72°C.

Amplifications of *mxaF*/*xoxF* genes were performed in 20 µL reactions using 8 µL of 2.5x 5 Prime Mastermix (MgCl_2_ final concentration 1.5 mM), 1.5 µL 3 % BSA (Biolab products GmbH, Lüneburg, Germany), 1 µL each of forward and reverse primers (10 µM; see Table 2 for primer sequences), 5 µL DNA template solution (between 1.5 - 15 ng), 0.5 µL distilled water, and 3 µL 5x TaqMaster PCR Enhancer (5 Prime GmbH, Hamburg, Germany). Two separate PCR reactions with two different forward primers mdh1 or mdh2 (Table S2) and the same reverse primer mdhR (Table 2 for primer sequences) were performed, yielding amplicons for both *mxaF* and *xoxF* types of methanol dehydrogenase genes. After initial denaturation (95°C, 5 min), DNA amplification was performed by 40 cycles of 1 min denaturation at 95°C, annealing for 1 min at 55°C, extension for 1 min at 72°C and a final extension step of 5 min at 72°C. PCR products of mdh1/mdhR and mdh2/mdhR amplicons were mixed prior to sequencing.

PCR products for taxonomical (16S rRNA) and functional (*cmuA* and *mxaF*/*xoxF*) genes were sequenced (LGC Genomics GmbH, Berlin, Germany). PCR fragments were barcoded. A total of 48 barcode sequences were used; 24 barcodes for the 16S PCR amplicons (eight microcosms, with two pooled fractions, i.e. heavy and light), and 24 for functional genes.

**Nucleic acid extraction from soil and RNA removal.** Nucleic acids from both duplicates of each of the 8 microcosms (0.5 g of soil) were extracted after CH_3_Cl had been consumed at the third substrate pulse (Griffiths *et al.*, 2000). Nucleic acids were resuspended in molecular biological grade water (30 µL), heat-treated for 3 min at 65°C prior to RNAs removal by incubating one-hour at 37°C with RnaseA (Sigma-Aldrich, Germany). DNA was precipitated with 21µL isopropanol and 6 µL 5 M NaCl overnight at -20°C. After centrifugation at 14,000×g for 1h at 4°C, the DNA pellet was washed with 70% (vol/vol) ethanol, air-dried at room temperature and resuspended in 30 µL RNase- and DNase-free water. DNA was quantified by using the Quant-iT™ PicoGreen® Kit (Invitrogen).

**DNA fractionation by isopycnic centrifugation.** Heavy (H) and light (L) DNA separation by a cesium chloride gradient was described in detail previously (Neufeld *et al.*, 2007b; Morawe *et al.*, 2017). In brief, the cesium chloride gradient solution mixed with each RNA-free DNA was loaded in an ultracentrifugation tube, placed in a Vti 65.2 vertical rotor (Beckman Coulter) and centrifuged on a LE-70 ultracentrifuge (Beckman Coulter) for 36 hours at 170,000×g. To harvest the DNA fraction without disrupting the cesium chloride gradient, a dye-containing water solution was injected from the top of the tube using a low-flow pump (BioRad Econo Pump) to push out DNA fractions which were collected with a sterile cannula that pierced the bottom of the tubes. Ten 450 µL-fractions were collected from each gradient tube and DNA from each fraction was precipitated as described (Neufeld *et al.*, 2007b), resuspended in 20 µL molecular-grade water. Four fractions of H and of L DNA solutions were pooled (Morawe *et al.*, 2017) and quantified with the Quant-iT™ PicoGreen® kit (Invitrogen). DNA concentrations ranged between 0.3 to 3.0 ng µL^-1^ for H fractions and 18.5 to 50.3 ng µL^-1^ for L fractions.

**Additional Information on PCR amplification, high-throughput sequencing, and data processing.** 24 barcodes for the 16S rRNA gene amplicons and 24 for functional genes using Illumina MiSeq technology by a commercial supplier (LGC Genomics GmbH, Berlin, Germany). Briefly, a barcode oligonucleotide identifying sequence was ligated to each PCR product. Equimolar pools of all libraries were assembled, and the resulting combined library was analyzed using a standard MiSeq paired end (2×300 bp) flow cell and reagent cartridge. Illumina reads were assembled into contigs and analyzed using the Mothur software package v.1.33.2 (Kozich *et al.*, 2013) with the default parameters of the MiSeq standard operating protocol (http://www.mothur.org/wiki/MiSeq_SOP).

**List 1. *cmuA*-like nucleotide database entries used to design the new primers cmuAf422 and cmuAr422**:

Alpha proteobacterium WG1,gi|66474962|gb|AY934501; Aminobacter_IMB-1,AF307143; Aminobacter_lissarensis CC495,AY838881; Aminobacter_TW23 methyltransferase, gi|66474964|gb|AY934502.1

bacterium_1,gi|12667208|gb|AF307140.1|AF307140:1-592

bacterium_2,gi|12667210|gb|AF307141.1|AF307141:1-592

bacterium_3,gi|12667212|gb|AF307142.1|

Desulfomonile_tiedjei DSM 6799,gi|390621545|gb|CP003360.1|:6269108-6270760

Desulfotomaculum alcoholivorax,WP_027363948

Desulfotomaculum kuznetsovii_DSM 6115, gb|CP002770.1|:61297-61962

Hyphomicrobiu_TW28,gi|66474956|gb|AY934498.1|:2-762

Hyphomicrobium_AT2,gi|343790367|emb|FN667868.1|:2225-4014

Hyphomicrobium_AT3,gi|343790372|emb|FN667869.1|:2213-4014

Hyphomicrobium_AT4,gi|343790376|emb|FN667870.1|:2213-4014

Hyphomicrobium_CM2, AF281259

Hyphomicrobium_LAT3,gi|66474954|gb|AY934497

Hyphomicrobium_MC1, FQ859181

Hyphomicrobium_SAC-1,gi|83721804|emb|AJ871015.1|:1-807

Hyphomicrobium_TW30,gi|66474952|gb|AY934496.1|:2-762

Hyphomicrobium_TW4,gi|66474958|gb|AY934499.1|:2-762

Hyphomicrobium_TW5,gi|66474960|gb|AY934500.1|:2-762

Methylobacterium_CM4 Mchl_5697

Rhodobacteraceae_179,gi|67008217|emb|AJ810826.1|:201-1993

Rhodobacteraceae_198,gi|67008223|emb|AJ810827.1|:265-2038

Thermincola_potens JR_TherJR_0143

Thermosediminibacter oceani DSM 16646_Toce_1533

Uncultured marine bacterium clone PML1A5,gi|74035817|emb|AJ810832.1|:1-764

Uncultured_AY934433.1|:1-764

Uncultured_E9.1,DQ090674

Uncultured_E9.22,gi|71919824|gb|DQ090673.1|:1-1337

Uncultured_E9.8,gi|71919814|gb|DQ090668.1|:1-1337

Uncultured_gi|42494407|gb|AY439205.1|:1-808

Uncultured_gi|42494409|gb|AY439206.1|:1-808

Uncultured_gi|66474834|gb|AY934428.1|:1-764

Uncultured_gi|66474838|gb|AY934430.1|:1-764

Uncultured_gi|66474840|gb|AY934431.1|:1-764

Uncultured_gi|66474842|gb|AY934432.1|:1-764

Uncultured_gi|66474846|gb|AY934434.1|:1-764

Uncultured_gi|66474848|gb|AY934435.1|:2-764

Uncultured_gi|66474850|gb|AY934436.1|:2-764

Uncultured_gi|66474854|gb|AY934438.1|:1-764

Uncultured_gi|66474856|gb|AY934439.1|:1-764

Uncultured_gi|66474858|gb|AY934440.1|:1-761

Uncultured_gi|66474860|gb|AY934441.1|:2-764

Uncultured_gi|66474866|gb|AY934444.1|:1-764

Uncultured_gi|66474870|gb|AY934446.1|:1-764

Uncultured_gi|66474874|gb|AY934448.1|:1-764

Uncultured_gi|66474876|gb|AY934449.1|:2-764

Uncultured_gi|66474878|gb|AY934450.1|:1-764

Uncultured_gi|66474880|gb|AY934451.1|:2-764

Uncultured_gi|66474882|gb|AY934452.1|:1-764

Uncultured_gi|66474884|gb|AY934453.1|:1-764

Uncultured_gi|66474886|gb|AY934454.1|:2-762

Uncultured_gi|66474888|gb|AY934455.1|:2-762

Uncultured_gi|66474892|gb|AY934457.1|:2-761

Uncultured_gi|66474894|gb|AY934458.1|:1-759

Uncultured_gi|66474896|gb|AY934459.1|:2-762

Uncultured_gi|66474898|gb|AY934460.1|:2-759

Uncultured_gi|66474902|gb|AY934462.1|:2-762

Uncultured_gi|66474904|gb|AY934463.1|:2-759

Uncultured_gi|66474906|gb|AY934464.1|:2-759

Uncultured_gi|66474910|gb|AY934466.1|:2-759

Uncultured_gi|66474912|gb|AY934467.1|:2-759

Uncultured_gi|66474914|gb|AY934468.1|:2-762

Uncultured_gi|66474916|gb|AY934469.1|:2-762

Uncultured_gi|66474918|gb|AY934470.1|:2-762

Uncultured_gi|66474920|gb|AY934471.1|:2-762

Uncultured_gi|66474922|gb|AY934472.1|:2-762

Uncultured_gi|66474924|gb|AY934473.1|:2-764

Uncultured_gi|66474928|gb|AY934475.1|:2-762

Uncultured_gi|66474930|gb|AY934476.1|:2-762

Uncultured_gi|66474932|gb|AY934477.1|:2-762

Uncultured_gi|66474934|gb|AY934478.1|:2-764

Uncultured_gi|66474936|gb|AY934479.1|:1-764

Uncultured_gi|66474938|gb|AY934480.1|:1-764

Uncultured_gi|66474940|gb|AY934481.1|:2-764

Uncultured_gi|66474942|gb|AY934482.1|:1-764

Uncultured_gi|66474944|gb|AY934483.1|:1-764

Uncultured_gi|66474946|gb|AY934484.1|:1-764

Uncultured_gi|66474948|gb|AY934485.1|:2-764

Uncultured_gi|66474950|gb|AY934486.1|:1-764

Uncultured_gi|71919808|gb|DQ090665.1|:1-1337

Uncultured_gi|71919810|gb|DQ090666.1|:1-1337

Uncultured_gi|71919812|gb|DQ090667.1|:1-1337

Uncultured_gi|71919816|gb|DQ090669.1|:1-1337

Uncultured_gi|71919818|gb|DQ090670.1|:1-1337

Uncultured_gi|71919820|gb|DQ090671.1|:1-1337

Uncultured_gi|71919822|gb|DQ090672.1|:1-1337

Uncultured_gi|71919827|gb|DQ090675.1|:2-764

Uncultured_gi|71919829|gb|DQ090676.1|:2-764

Uncultured_gi|71919831|gb|DQ090677.1|:2-764

Uncultured_gi|71919835|gb|DQ090679.1|:2-764

Uncultured_gi|71919837|gb|DQ090680.1|:2-764

Uncultured_gi|71919839|gb|DQ090681.1|:2-764

Uncultured_gi|71919841|gb|DQ090682.1|:2-764

Uncultured_gi|71919843|gb|DQ090683.1|:2-764

Uncultured_gi|71919847|gb|DQ090685.1|:13-764

Uncultured_gi|71919848|gb|DQ090686.1|:13-767

Uncultured_gi|71919850|gb|DQ090687.1|:13-767

Uncultured_gi|71919854|gb|DQ090689.1|:13-767

Uncultured_gi|71919856|gb|DQ090690.1|:13-767

Uncultured_gi|71919870|gb|DQ090697.1|:2-764

Uncultured_gi|71919875|gb|DQ090700.1|:13-767

Uncultured_gi|71919877|gb|DQ090701.1|:13-767

Uncultured_gi|71919879|gb|DQ090702.1|:13-767

Uncultured_gi|71919881|gb|DQ090703.1|:13-767

Uncultured_gi|71919883|gb|DQ090704.1|:2-764

Uncultured_gi|71919885|gb|DQ090705.1|:13-767

Uncultured_gi|74035815|emb|AJ810831.1|:1-764

Uncultured_gi|74035819|emb|AJ810833.1|:1-764

Uncultured_MeBr 12,gi|42494413|gb|AY439208.1|:1-808

**List 2. *mxaF/xoxF* nucleotide database entries used to design the new forward primers mdh1 and mdh2 and the reverse primer mdhR**:

*Acidomonas methanolica* strain MB58 (gi|546198649|dbj|AB855793.1 mxaF)

*Afipia felis* strain 25E-1 (gi|56967356|gb|AY848826.1 mxaF) and strain RD1 (gi|56967358|gb|AY848827.1 mxaF)

*Azospirillum halopraeferens* strain DSM 3675 (AUCF_v1_310023 mxoF; AUCF_v1_390016 xoxF)

*Azospirillum lipoferum* strain 4B (AZOLI_p10948 mxoF1; AZOLI_p10703 mxoF2)

*Beijerinckia mobilis* strain DSM 2326 (gi|60457989|emb|AJ563936.2 mxaF)

*Burkholderiales* bacterium RZ18-153 (gi|177826803|gb|EU548067.1 mxa)

*Enterobacter arachidis* strain Ah-143 (gi|221327284|gb|EU912491.1 mxaF)

*Granulibacter bethesdensis* strains CGDNIH1 (gi|114314838|gb|CP000394.1|:393505-395281 xoxF5; gi|114314838|gb|CP000394.1|:2155943-2157555 xoxF5; gi|114314838|gb|CP000394.1|:736249-737277 mxaF); strain CGDNIH2 (gi|586605219|gb|CP003183.1|:393525-395301; gi|586605219|gb|CP003183.1|:1273794-1275406; gi|586605219|gb|CP003183.1|:736068-737096); strain CGDNIH3 (gi|586599970|gb|.1|:395617-397393; gi|586599970|gb|CP003181.1|:738881-739909) and strain CGDNIH4 (CP003182.1; gi|586602595|gb|CP003182.1|:1281176-1282788; gi|586602595|gb|CP003182.1|:742703-743731; gi|586602595|gb|CP003182.1|:138959-139797)

*Hyphomicrobium denitrificans* ATCC 51888 (Hden_1323 MxaF; Hden_1869 XoxF5; Hden_1617 XoxF5; Hden_1305 XoxF5; Hden_2848 XoxF5)

*Hyphomicrobium methylovorum* (ENA|BAA23272|BAA23272.1 ; gi|2588996|dbj|AB004097.1|:285-2070)

*Hyphomicrobium nitrativorans* NL23 (W911_13290 xoxF; W911_14545 mxaF)

*Hyphomicrobium* sp. MC1 (HYPMCv2_0485 mxaF; HYPMCv2_4120 xoxF5; HYPMCv2_1896 xoxF; HYPMCv2_0800 xoxF5; HYPMCv2_3613 xoxF3)

*Hyphomicrobium zavarzinii* (gi|2398663|emb|Y08080.1 mxaF gene)

*Methylobacillus flagellatus* KT (Mfla_2044 mxaF; Mfla_0344 xoxF4; Mfla_2314 xoxF4; Mfla_1717 xoxF4; Mfla_1451 xoxF3)

*Methylobacter luteus* IMV-B-3098T (MLUTv2_12038 mxaF; MLUTv2_10236 xoxF; MLUTv2_11346 xoxF)

*Methylobacterium aerolatum* strain KACC11766 (gi|218533636|gb|EU912496.1 mxaF)

*Methylobacterium aminovorans* (gi|166079885|gb|EU194911.1 mxaF)

*Methylobacterium extorquens* strain AM1 (META1_4538 mxaF; META1_2757 xoxF; META1_1740 xoxF5) ; strain BJ001 (MEXB_5194 mxaF; MEXB_1975 xoxF5) ; strain CM4 (Mchl_4518 mxaF; Mchl-2145 xoxF5; Mchl_5444, xoxF5) ; strain DM4 (METDI5145 mxaF; METDI2492 xoxF5) ; strain PA1 (Mext_4150 mxaF; Mext_1809 xoxF5; Mext_0099 xoxF3)

*Methylobacterium lusitanum* strain KCTC 12964 (gi|156539224|gb|EF562469.1 mxaF) ; strain MP2 (gi|118639596|gb|EF030548.1 mxaF) ; strain NBAIMmx4 (gi|317375435|gb|HM765481.1 mxaF-like)

*Methylobacterium mesophilicum* SR1.6/6 (ANPAv1_180157 MxaI ; ANPAv1_150222 xoxF5; ANPAv1_30068 xoxF5)

*Methylobacterium nodulans* strain ORS2060 (Mnod_8040 mxaF; Mnod_0421 xoxF5; Mnod_2344 xoxF3; Mnod_5977 xoxF5)

*Methylobacterium organophilum* (M22629.1) ; strain NRIC 0602 (gi|224471287|dbj|AB455975.1 mxaF)

*Methylobacterium oryzae* (ENA|ABL09521|ABL09521.1)

*Methylobacterium phyllosphaerae* (ENA|ABU80565|ABU80565.1)

*Methylobacterium podarium* (gi|320117110|gb|HQ588895.1 mxaF)

*Methylobacterium podarium* strain FM4 (gi|40716505|gb|AY468366.1 mxaF)

*Methylobacterium populi* strain NBAIMmx8 (gi|317375439|gb|HM765485.1 mxaF)

*Methylobacterium radiotolerans* JCM 2831 (Mrad2831_4210 mxaF; Mrad2831_0508 xoxF5; Mrad2831_1932 xoxF5)

*Methylobacterium rhodesianum* strain DSM 5687 (gi|156539229|gb|EF562473.1)

*Methylobacterium rhodinum* (gi|2326973|gb|U70527.1 mxaF)

*Methylobacterium salsuginis* (320117115|gb|HQ588898.1 mxaF) ; strain MR (gi|118639600|gb|EF030550.1 mxaF); strain NBHP13 (gi|319998990|gb|HQ221369.1 mxaF)

*Methylobacterium* sp. strain AR1.6/2 (gi|197210514|gb|EU789423.1) ; strain CBMB38 (gi|156539244|gb|EF562482.1 mxaF) ; strain II_PPFM_10 (gi|306476495|gb|HQ219738.1 mxaF) ; strain II_PPFM_2 (gi|306476509|gb|HQ219745.1 mxaF); strain II_PPFM_3 (gi|306476511|gb|HQ219746.1 mxaF) ; strain II_PPFM_7 (gi|306476507|gb|HQ219744.1 mxaF) ; strain MB200 (AMXUv1_180118 MxaF; AMXUv1_460164) ; strain MNW60 (gi|294997381|gb|GU980953.1 mxaF); strain MP3 (ENA|ABL09523|ABL09523.1) ; strain MSF32 (gi|294997379|gb|GU980952.1 mxaF); strain NBAIMmx6 (gi|317375437|gb|HM765483.1 mxaF); strain SR3/27 (gi|197210512|gb|EU789434.1 mxaF); strain TC3-6 (gi|197210456|gb|EU789407.1 mxaF) ; strain TP4-1 (gi|197210481|gb|EU789441.1 mxaF-like)

*Methylobacterium suomiense* strain CBMB120 (gi|156539236|gb|EF562477.1 mxaF); strain CBMB130 (gi|156539240|gb|EF562479.1 mxaF); strain KCTC 12963 (gi|156539231|gb|EF562474.1 mxaF)

*Methylobacterium thiocyanatum* strain DSM 11490 (gi|156539233|gb|EF562475.1 mxaF)

*Methylobacterium zatmanii* strain DSM 5688 (gi|116876195|gb|EF031553.1 mxaF); strain KACC11431 (gi|156539214|gb|EF562463.1 mxaF); strain NBAIMmx16 (gi|317375447|gb|HM765493.1 mxaF)

*Methyloceanibacter caenitepidi* strain Gela4 (AB844438.1 mxaF)

*Methylocella silvestris* strain BL2 (Msil_0471 mxaF; Msil_1587; Msil_3387 xoxF3; Msil_2260)

*Methylococcus capsulatus* strain Bath (MCA0779 mxaF; MCA0299 xoxF5)

*Methylocystis* sp. ATCC 4924 (MSPATv1_180053 mxaF; MSPATv1_10147 xoxF1; MSPATv1_130043 xoxF2)

*Methylomicrobium alcaliphilum* strain 20Z (MALCv4_3448 mxaF; MALCv4_3497 xoxF5)

*Methylomicrobium* sp. HG-1 (gi|92430142|gb|DQ463162.1 mxa)

*Methylophilus methylotrophus* (ENA|U41040|U41040.1)

*Methylopila capsulata* strain ATCC 700716 (gi|62996383|emb|AJ878071.1 mxaF)

*Methylorhabdus multivorans* (gi|62996381|emb|AJ878070.1 mxaF)

*Methylosinus* sp. B4S (AB741958.1 mxaF)

*Methylosinus trichosporium* OB3b (MTRIv1_240014 mxaF)

*Methyloversatilis universalis* FAM5 (METUNv2_590218 xoxF; METUNv2_590217 xoxF)

*Paracoccus denitrificans* (ENA|AAC44555|AAC44555.1; ENA|AAA88366|AAA88366.1); strain PD1222 (gi|119376152|gb|CP000490.1|:166925-168706)

*Pedobacter* sp. strain A48 (gi|183241343|gb|EU439313.1)

*Rhodopseudomonas palustris* strain BisB18 (gi|90103542|gb|CP000301.1; gi|115515977|gb|CP000463.1)

*Starkeya* novella strain DSM 506 (gi|296926528|gb|CP002026.1|:4425523-4427305; gi|296926528|gb|CP002026.1|:1073754-1075361)

*Verminephrobacter eiseniae* strain EF01-2 (gi|121551644|gb|CP000542.1|:5502519-5504136; gi|121551644|gb|CP000542.1|:2041924-2043578)

Uncultured bacterium 16A2 (gi|170762577|gb|EU362857.1 mxaF); strain CBMB204 (EU439311.1); strain CBMB205 (EU439312.1)

Uncultured *Methylobacterium* sp. clone NBAIMmetamx2 (gi|319998959|gb|HM628892.1 mxaF); clone: SH14-21 (gi|402244197|dbj|AB683122.1 mxaF); clone: SM14-28 (gi|402244213|dbj|AB683130.1 mxaF); clone: SM24-23 (gi|402244203|dbj|AB683125.1 mxaF); clone: SM41-21 (gi|402244215|dbj|AB683131.1 mxaF)

uncultured *Methylophaga* sp. (ENA|ABS82782|ABS82782.1)

Uncultured methylotrophic bacterium clone E8RTMAX20 (gi|84621410|gb|DQ260411.1 mxaF); clone G11RTMAX475 (gi|84621405|gb|DQ260406.1 mxaF); clone G9RTMXAA93 (gi|84621406|gb|DQ260407.1 mxaF); clone NT (gi|51511948|gb|AY686551.1 mxaF); clone NTg (gi|51511950|gb|AY686552.1 mxaF); clone VTg (gi|51511952|gb|AY686553.1 mxaF) and clone wp8 (gi|30724787|emb|AJ561092.1 mxaF)

**References**

Borodina E, Cox MJ, McDonald IR, Murrell JC. (2005). Use of DNA-stable isotope probing and functional gene probes to investigate the diversity of methyl chloride-utilizing bacteria in soil. *Environ Microbiol* **7:** 1318-1328.

Crooks GE, Hon G, Chandonia JM, Brenner SE. (2004) WebLogo: a sequence logo generator. *Genome Res* **14**:1188-1190.

Farhan Ul Haque M, Besaury L, Nadalig T, Bringel F, Mutterer J, Schaller H *et al.* (2017). Correlated production and consumption of chloromethane in the *Arabidopsis thaliana* phyllosphere. *Sci Rep* **7:** 17589.

Griffiths RI, Whiteley AS, O'Donnell AG, Bailey MJ. (2000). Rapid method for coextraction of DNA and RNA from natural environments for analysis of ribosomal DNA- and rRNA-based microbial community composition. *Appl Environ Microbiol* **66:** 5488-5491.

Keltjens JT, Pol A, Reimann J, Op den Camp HJ. (2014). PQQ-dependent methanol dehydrogenases: rare-earth elements make a difference. *Appl Microbiol Biotechno*l **98:** 6163–6183.

Kozich JJ, Westcott SL, Baxter NT, Highlander SK, Schloss PD. (2013). Development of a dual-index sequencing strategy and curation pipeline for analyzing amplicon sequence data on the miSeq Illumina sequencing platform. *Appl Environ Microbiol* **79:** 5112-5120.

Larkin MA, Blackshields G, Brown NP, Chenna R, McGettigan PA, McWilliam H *et al*.
(2007). [Clustal W and Clustal X version 2.0.](http://www.ncbi.nlm.nih.gov/pubmed/17846036) *Bioinformatics* **23:** 2947-2948.

McDonald IR, Murrell JC. (1997). The methanol dehydrogenase structural gene *mxaF* and its use as a functional gene probe for methanotrophs and methylotrophs. *Appl Environ Microbiol* **63:** 3218-3224.

Miller LG, Warner KL, Baesman SM, Oremland RS, McDonald IR, Radajewski S *et al.* (2004). Degradation of methyl bromide and methyl chloride in soil microcosms: Use of stable C isotope fractionation and stable isotope probing to identify reactions and the responsible microorganisms. *Geochim Cosmochim Acta* **68:** 3271-3283.

Moosvi SA, McDonald IR, Pearce DA, Kelly DP, Wood AP. (2005). Molecular detection and isolation from Antarctica of methylotrophic bacteria able to grow with methylated sulfur compounds. *Syst Appl Microbiol* **28:** 541-554.

Morawe M, Hoeke H, Wissenbach DK, Lentendu G, Wubet T, Kröber E *et al.* (2017). Acidotolerant bacteria and fungi as a sink of methanol-derived carbon in a deciduous forest soil. *Front Microbiol* **8:** 1361.

Morris SA, Radajewski S, Willison TW, Murrell JC. (2002). Identification of the functionally active methanotroph population in a peat soil microcosm by stable-isotope probing. *Appl Environ Microbiol* **68:** 1446-1453.

Nadalig T, Farhan Ul Haque M, Roselli S, Schaller H, Bringel F, Vuilleumier S. (2011). Detection and isolation of chloromethane-degrading bacteria from the *Arabidopsis thaliana* phyllosphere, and characterization of chloromethane utilisation genes. *FEMS Microbiol Ecol* **77:** 438-448.

Nadalig T, Greule M, Bringel F, Keppler F, Vuilleumier S. (2014). Probing the diversity of chloromethane-degrading bacteria by comparative genomics and isotopic fractionation. *Front Microbiol* **5**:523.

Neufeld JD, Schäfer H, Cox MJ, Boden R, McDonald IR, Murrell JC. (2007a). Stable-isotope probing implicates *Methylophaga* spp and novel Gammaproteobacteria in marine methanol and methylamine metabolism. *ISME J* **1:** 480-491.

Neufeld JD, Vohra J, Dumont MG, Lueders T, Manefield M, Friedrich MW *et al.* (2007b). DNA stable-isotope probing. *Nature Prot* **2:** 860-866.

Radajewski S, Ineson P, Parekh NR, Murrell JC. (2000). Stable-isotope probing as a tool in microbial ecology. *Nature* **403:** 646-649.

Radajewski S, Webster G, Reay DS, Morris SA, Ineson P, Nedwell DB *et al.* (2002). Identification of active methylotroph populations in an acidic forest soil by stable-isotope probing. *Microbiology* **148:** 2331-2342.

Stacheter A, Noll M, Lee CK, Selzer M, Glowik B, Ebertsch L *et al.* (2013). Methanol oxidation by temperate soils and environmental determinants of associated methylotrophs. *ISME J* **7:** 1051-1064.

Taubert M, Grob C, Howat AM, Burns OJ, Dixon JL, Chen Y *et al.* (2015). *Xoxf* encoding an alternative methanol dehydrogenase is widespread in coastal marine environments. *Environ Microbiol* **17:** 3937-3948.
